# A cross-sectional study assessing the association between online ratings and structural and quality of care measures: results from two German physician rating websites

**DOI:** 10.1186/s12913-015-1051-5

**Published:** 2015-09-24

**Authors:** Martin Emmert, Thomas Adelhardt, Uwe Sander, Veit Wambach, Jörg Lindenthal

**Affiliations:** Friedrich-Alexander-University Erlangen-Nuremberg, School of Business and Economics, Institute of Management (IFM), Lange Gasse 20, 90403 Nuremberg, Germany; Chair of Health Management, Friedrich-Alexander-University Erlangen-Nuremberg, School of Business and Economics, Institute of Management (IFM), Lange Gasse 20, 90403 Nuremberg, Germany; University of Applied Sciences and Arts, Hannover, Germany; Integrated Healthcare Network “Quality and Efficiency” (QuE eG), Nuremberg, Vogelsgarten 1, 90402 Nuremberg, Germany

**Keywords:** Physician rating website, Patient experience, Internet, Quality of care

## Abstract

**Background:**

Even though physician rating websites (PRWs) have been gaining in importance in both practice and research, little evidence is available on the association of patients’ online ratings with the quality of care of physicians. It thus remains unclear whether patients should rely on these ratings when selecting a physician. The objective of this study was to measure the association between online ratings and structural and quality of care measures for 65 physician practices from the German Integrated Health Care Network “Quality and Efficiency” (QuE).

**Methods:**

Online reviews from two German PRWs were included which covered a three-year period (2011 to 2013) and included 1179 and 991 ratings, respectively. Information for 65 QuE practices was obtained for the year 2012 and included 21 measures related to structural information (*N* = 6), process quality (*N* = 10), intermediate outcomes (*N* = 2), patient satisfaction (*N* = 1), and costs (*N* = 2). The Spearman rank coefficient of correlation was applied to measure the association between ratings and practice-related information.

**Results:**

Patient satisfaction results from offline surveys and the patients per doctor ratio in a practice were shown to be significantly associated with online ratings on both PRWs. For one PRW, additional significant associations could be shown between online ratings and cost-related measures for medication, preventative examinations, and one diabetes type 2-related intermediate outcome measure. There again, results from the second PRW showed significant associations with the age of the physicians and the number of patients per practice, four process-related quality measures for diabetes type 2 and asthma, and one cost-related measure for medication.

**Conclusions:**

Several significant associations were found which varied between the PRWs. Patients interested in the satisfaction of other patients with a physician might select a physician on the basis of online ratings. Even though our results indicate associations with some diabetes and asthma measures, but not with coronary heart disease measures, there is still insufficient evidence to draw strong conclusions. The limited number of practices in our study may have weakened our findings.

**Electronic supplementary material:**

The online version of this article (doi:10.1186/s12913-015-1051-5) contains supplementary material, which is available to authorized users.

## Background

Physician rating websites (PRW) have become a popular tool for increasing transparency regarding the quality of care of physicians in the outpatient sector [1–5]. One intention of PRWs is to provide information regarding patient satisfaction to enable patients to make an informed choice when selecting a physician. Besides a scaled survey, most PRWs implement a free commentary field [6] so that patients can report on their experience without any constraints other than word limitation. So far, studies have shown the increasing popularity of such websites when it comes to the number of ratings [2, 7, 8], the traffic rank [8, 9], and the awareness of the population [10], and others have addressed the content and nature of narrative comments [3, 11, 12] or analyzed the applied patient satisfaction surveys [6]. One result is that a large proportion of online ratings is positive in the countries analyzed, such as the USA [2, 5, 12–15], the UK [16], Germany [8, 9], and Canada [17].

However, it remains unclear whether patients should rely on the ratings displayed on such sites when choosing a physician [1]. Selecting a physician on the basis of online ratings would increase the likelihood of receiving better healthcare provision. Greaves and colleagues showed in a pioneer study a relationship between online hospital ratings and objective measures of clinical quality in the UK. They concluded that patients who base their decision on this information can be assured that the ratings are not entirely misleading and may provide relevant information about health care [18]. However, whether patients should base their selection on online ratings for a physician in the outpatient sector remains less clear [16].

In this context, the present study aims at adding further knowledge on whether patient satisfaction results displayed on PRWs demonstrate an association with structural and quality of care measures of healthcare providers. This will allow for an analysis of the value of online ratings for patients searching for a physician online.

## Methods

### Structural and quality of care measures

We received structural and quality of care data for 65 physician practices from the German Integrated Health Care Network “Quality and Efficiency” (QuE) for the year 2012 [19, 20]. Thereby, 32 general practices and 33 specialist practices were included (no dentists). All data were provided on an aggregated practice level. In total, the data set included 21 measures which could be assigned to five categories. First, six measures provided structural information on the physicians and their practice (e.g., the number of physicians per practice, the age of the physicians, and the number of patients per practice). Second, seven process quality measures were provided for three chronic diseases (diabetes type 2, coronary heart disease, and asthma). Two further measures related to medication therapy in the elderly and addressed polypharmacy and potentially inappropriate medications by applying the PRISCUS list [21] (the latter carry an increased risk of adverse drug events in the elderly; the data for the latter were dated from 2011). In addition, one preventative examination measure was included. Third, two diabetes type 2-related intermediate outcome measures were included. Fourth, an offline patient survey was conducted in October 2012 to assess patient satisfaction in the 65 practices. In total, 4553 patients completed the survey (response rate 56.9 %). The rating system in German schools was applied, which ranges from (1) = very good to (6) = insufficient. We selected the overall score of the survey as a measure. Fifth, two measures indicated the costs of medication therapy from the perspective of German health funds. One measure shows the costs per prescribed medication per case and the other refers to the costs per prescription.

### Online ratings data

We obtained all aggregated online ratings for the 65 QuE practices displayed on the PRWs jameda (*N* = 1179) and the Weisse Liste (*N* = 991) for a three-year period (2011 to 2013) from the provider of the websites. The rating system on jameda consists of five mandatory questions, rated according to the grading system in German schools, from (1) = very good to (6) = insufficient. The questions refer to: (Q1) satisfaction with the treatment offered; (Q2) information and presentation of facts with regard to illness and treatment; (Q3) the relationship of trust with the physician; (Q4) the amount of time spent on a patient’s concerns, and (Q5) the friendliness of the physician. An average score is calculated on the basis of the five single grades. Beyond that, a narrative commentary has to be given and 13 optional questions are available for answering [6]. In order to leave a rating on jameda, a user has to register on the website.

In contrast, on the Weisse Liste, only those insured by four large German health funds (Allgemeine Ortskrankenkassen, BARMER GEK, Techniker Krankenkasse, and Bertelsmann Betriebskrankenkasse) and who are 15 years of age or older can rate a doctor. They therefore need to register with the data shown on their insurance card to prevent the manipulation of ratings. Interestingly, a minimum of five ratings for physicians is publicly displayed [22]. The survey comprises 33 questions related to the four dimensions of practice and staff (13 questions), communication (7 questions), treatment (9 questions), and overall impression (4 questions). For our analysis, we focused on the latter dimension since the questions are more likely to provide an overall impression of the care received. Here, the questions relate to the overall impression of the physician, the experience of the treatment received, whether one would recommend the physician to one’s best friend, and whether one would visit the physician again for further treatment [23]. The ratings are made on a scale of one (lowest rating) to five (highest rating).

### Statistical analysis

All statistical analyses of the data were carried out by means of SPSS v21.0 (IBM Corp, Armonk, NY, USA). Descriptive analysis included calculating the mean, standard deviation (SD), minimum and maximum for all QuE measures. The Spearman rank coefficient of correlation was applied to measure the association between online ratings and practice-related structural and quality of care information since none of our dependent variables from both PRWs were normally distributed according to the Shapiro-Wilk test (*p* < 0.001, data not shown here). Differences were considered to be significant if *p* < .05, and highly significant if *p* < .001.

### Systematic search procedure to identify comparable literature

We conducted a systematic search procedure on Medline (via PubMed) to identify studies which addressed a similar question to ours. The search was carried out in October 2014 and aimed at identifying English language literature published since 2009. This timeframe was chosen since the topic of physician rating websites is still a relatively young area of research. The search terms applied included terms regarding patient experience (e.g., patient experience, patient opinion, patient rating, patient satisfaction, consumer satisfaction) and quality of care (e.g., healthcare outcome, quality of care, quality outcomes). Those terms were derived from other systematic reviews which addressed either the topic of patient experience (e.g., [24–26]) or the quality of care (e.g., [27–29]).

### Ethical approval

No formal application to the Ethics Committee is mandatory for this investigation according to the appraisal of the Ethics Committee of the Friedrich-Alexander University of Erlangen-Nuremberg, Germany.

## Results

An overview of the structural and quality measures for all 65 physician practices is provided in Table 1. The mean number of physicians per practice was 1.77 (SD 1.09) and the mean age of all physicians was calculated to be 53.40 years (SD 5.29). In total, 991 ratings from the PRW Weisse Liste and 1179 ratings from the PRW jameda were included in our analysis. The mean number of ratings per practice was 16.80 (SD 17.63) and 19.98 (SD 24.15), respectively. The maximum number of ratings for one practice was 72 on the Weisse Liste and 157 on jameda, respectively. As shown in Table 2, the ratings for the 65 practices were positive overall. On the Weisse Liste, the ratings ranged between 3.92 and 4.45 on a scale of one (lowest) to five (highest), and those on jameda ranged between 1.56 and 1.76 on a scale of one (highest) to six (lowest).

**Table 1:** Descriptive analysis of the structural and quality measures for the 65 physician practices

Structural information			N	Mean	SD	Min	Max
1		Number of physicians per practice	65	1.77	1.09	1	8
2		Age of the physicians per practice (average)	63	53.40	5.29	42	65
3		Patients per practice per quarter (average)	58	1293.85	1019.28	41	6481.67
4		Patient per doctor ratio	58	774.53	382.59	20.50	1969.50
5		Quality circle visits (values practice-related)	65	8.42	3.12	0	16
6		Chronically ill patients (Q4/2012) (percentage)	32	83.68	17.16	32.00	100.00
Process quality measures			N	Mean	SD	Min	Max
7	Diabetes type 2	Patients with a diabetic retinal exam within the last 12 months (percentage)^b^	29	77.72	32.39	0.00	100.00
8	Diabetes type 2	Patients who had an ophthalmological examination in 2012 (percentage)^b^	24	42.78	11.34	18.60	70.00
9	Coronary heart disease	Patients who have been prescribed antiplatelet agents (percentage)^b^	29	88.48	23.19	10.00	100.00
10	Coronary heart disease	Patients who have been prescribed beta-blockers (percentage)^b^	29	85.61	12.21	62.50	100.00
11	Coronary heart disease	Patients with cardiac insufficiency who have been prescribed ACE inhibitors (percentage)^b^	28	74.21	23.79	25.00	100.00
12	Coronary heart disease	Patients who have been prescribed CHD statins (percentage)^b^	29	75.74	23.73	0.00	100.00
13	Asthma	Patients with long-term medication who have been prescribed inhaled corticosteroids (percentage)^b^	26	83.24	20.46	40.00	100.00
14	Medication in the elderly	Polypharmacy: Patients aged 65 years or older with more than eight prescribed medications (Q4/2012) (percentage) [source Sickness Fund]	32	9.50	5.26	0.00	20.90
15	Medication in the elderly	PRISCUS medication (2011) (percentage) [source Sickness Fund]^c^	60	2.50	1.36	1.00	5.00
16	Prevention	Patients aged 35 or older with a general preventive examination (percentage)^b^	32	58.47	22.28	6.72	95.95
Intermediate outcome measures			N	Mean	SD	Min	Max
17	Diabetes type 2	Patients who reached individual HbA1c-target values (percentage)^b^	29	73.97	19.32	25.53	100.00
18	Diabetes type 2	Patients with hypertension who show a normotensive blood pressure (percentage)^b^	29	63.40	17.72	31.01	98.77
Patient satisfaction			N	Mean	SD	Min	Max
19		Offline patient survey 2012 (practice-related)	52	1.39	0.14	1.05	1.69
Medication prescription: cost related measures			N	Mean	SD	Min	Max
20		Cost per case (average 2012)^a^	62	85.31	69.71	1.25	382.72
21		Cost per prescription (average 2012)^a^	62	44.53	42.92	16.44	309.26

^a^This indicator is based on claims data

^b^This indicator applies only to the 32 general practices

^c^The data shown refer to groups (group 1 < =2 %, group 2 < =5 %, group 3 < =7.5 %, group 4 < =10 %, group 5 > 10 %)

**Table 2:** Descriptive analysis of the online ratings for the 65 physician practices on both PRWs

Distribution of the number of ratings			Mean	SD	Min	Max	Sum
	Weisse liste		16.80	17.63	1	72	991
	jameda		19.98	24.15	1	157	1179
Rating results			Mean	SD	Min	Max	N
	Weisse liste (5 = highest, 1 = lowest)						
		Q30: What is your overall impression of the physician?	4.01	0.93	1.00	5.00	59
		Q31: How would you describe the experience of the received treatment?	3.92	0.84	1.00	5.00	59
		Q32: Would you recommend the physician to your best friend?	4.30	1.02	1.00	5.00	59
		Q33: Would you visit the physician again for further treatment?	4.45	1.04	1.00	5.00	59
	jameda (1 = highest, 6 = lowest)						
		Overall performance	1.68	0.60	1.00	3.46	59
		Q1: Satisfaction with the treatment by the physician	1.68	0.62	1.00	3.50	59
		Q2: Education about the illness and treatment	1.70	0.61	1.00	3.43	59
		Q3: Relationship of trust with the physician	1.72	0.68	1.00	3.75	59
		Q4: Time the physician spent on the patient’s concerns	1.76	0.67	1.00	3.86	59
		Q5: Friendliness of the physician	1.56	0.53	1.00	3.14	59

### Bivariate analysis of online ratings from the Weisse Liste and structural and quality measures

Significant associations could be determined for 6 of the 21 measures in total (see Table 3 and Additional file 1). Regarding the structural information, the patient per doctor ratio was significantly negatively associated with all four analyzed questions (Spearman *p* = −0.289 – −0.332, *p* < 0.05 for all). A further significant association was measured between the question of whether one would visit the physician again for further treatment and one process (patients aged 35 or older received a general preventative examination; *p* = 0.386, *p* < 0.05), and one intermediate outcome measure (diabetes type 2 patients who achieved individual HbA1c-target values; *p* = 0.478, *p* < 0.05). The results from the online ratings and the offline survey proved to be significantly associated in three of the four measures (*p* = −0.347 – −0.372, *p* < 0.05 for all). Finally, regarding the cost-related measures for medication prescription, the cost per case was significantly associated three times (*p* = 0.297 – 0.384, *p* < 0.05 for all), and the cost per prescription was associated with one question (*p* = 0.264, *p* < 0.05).

**Table 3:**
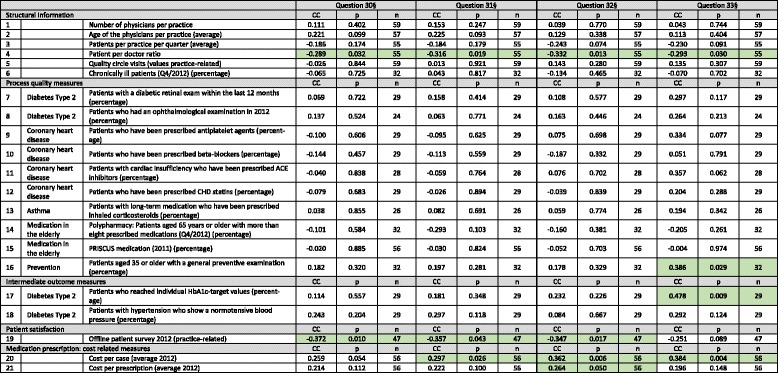
Bivariate analysis of online ratings from the PRW Weisse Liste and structural and quality measures (Spearman rank coefficient of correlation) [significant associations are highlighted in light green]

^**§**^Q30: How is your overall impression of the physician? Q31: How would you describe the experience of the received treatment? Q32: Would you recommend the physician to your best friend? Q33: Would you visit the physician again for further treatment? [The ratings are to be made on a 1 (lowest rating) to 5 (highest rating) scale]

### Bivariate analysis of online ratings from jameda and structural and quality measures

Seven out of the 21 measures were significantly associated (see Table 4 and Additional file 1). First, three structural measures were proven to be associated, namely the number of patients per practice per quarter (*p* = 0.294 – 0.350, *p* < 0.05 for all) and the patient per doctor ratio (*p* = 0.298 – 0.386, *p* < 0.05 for all) for all six measures. Here again, the ages of the physicians per practice were shown to be associated for two measures (*p* = 0.263 – 0.273, *p* < 0.05 for all). Regarding the process quality measures, one diabetes type 2 (patients who had an ophthalmological examination in 2012; *p* = −0.540 – -0.468, *p* < 0.05 for all) and one asthma indicator (patients with long-term medication prescribed inhaled corticosteroids; *p* = −0.552 – −0.435, *p* < 0.05 for all) were significantly negatively associated with four out of the six measures. All six jameda measures were associated with the offline survey (*p* = −0.391 – 0.640, *p* < 0.05 for all). Finally, one cost-related measure for medication prescription was also associated with the online ratings (cost per case; *p* = −0.298, *p* < 0.05).

**Table 4:**
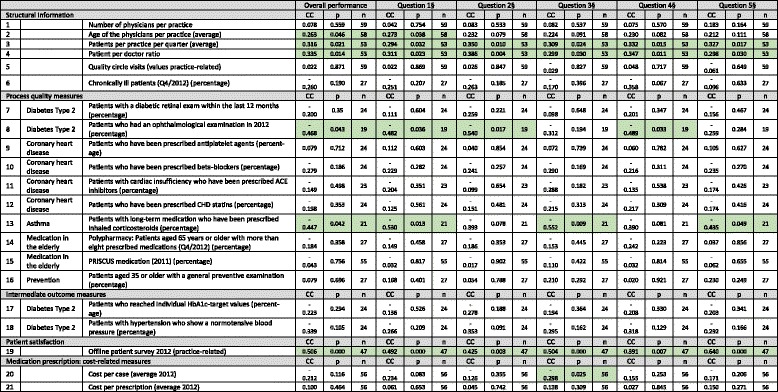
Bivariate analysis of online ratings from the PRW jameda and structural and quality measures (Spearman rank coefficient of correlation) [significant associations are highlighted in light green]

^**§**^Q1: Satisfaction with the treatment by the physician, Q2: Education about the illness and treatment, Q3: Relationship of trust with the physician, Q4: Time the physician spent on the patient’s concerns, Q5: Friendliness of the physician

## Discussion

The question of whether or not there is an association between patient satisfaction in general and the quality of care of the healthcare provider is not new. For example, a recently published systematic review explored the evidence on the links between patient satisfaction and clinical safety and effectiveness outcomes and included 55 studies in the analysis. The authors demonstrated positive associations between patient experience and self-rated and objectively measured health outcomes, adherence to recommended clinical practice and medication, preventative care, and resource use. However, this result is based on studies carried out with several criteria, such as the exclusion of studies with fewer than 50 subjects, or the use of validated survey instruments (e.g., Picker surveys, the Hospital Consumer Assessment of Healthcare Providers and Systems survey) [30]. In contrast, these preconditions were not fulfilled for the case of PRWs, since the majority of rated physicians on such sites is still far below this threshold [2, 7, 8] and most instruments measuring satisfaction have not been validated. Furthermore, although traditional surveys have the advantage of random allocation, respondents on PRWs offer unsolicited opinions, particularly when they have experienced extremes of care [31]. It thus remains questionable whether the above-mentioned association applies to online ratings and, consequently, whether patients should rely on these ratings when selecting a physician.

We assessed the association between online ratings and several structural and quality of care measures for a sample of 65 physician practices. Compared with previously published results for Germany [8, 9], the relatively high average number of ratings per physician could be attributed to some Weisse Liste advertisements in QuE reports, discussion of PRWs in general in quality circles, and other QuE events that might have led to higher awareness and use levels [20]. Some studies with a similar research question were identified by means of our systematic search procedure (see above) [16, 18, 31, 32]. First, one study from the UK examined hospital-level associations between 10,274 web-based patient ratings displayed on the NHS Choices website and indicators of clinical outcomes as well as healthcare-acquired infections of all NHS acute hospital trusts in England [18]. The positive recommendations of hospitals on NHS Choices were significantly associated with lower standardized mortality ratios, lower mortality from high-risk conditions, and lower readmission rates. Both healthcare-acquired infection measures were significantly associated with the online rating of hospital cleanliness. In another study, Greaves and colleagues analyzed the associations between internet-based patient ratings and conventional surveys of patient experience in England [31]. Web-based ratings for 146 hospitals displayed on NHS Choices (*N* = 9997) were compared with five similar questions from a national paper-based survey. As a result, statistically significant associations were demonstrated for all questions (*p* = 0.31 – 0.49, *p* < 0.001 for all). The third study assessed the relationship between website ratings from Yelp.com and traditional hospital performance measures in the USA [32]. The latter included patient experience (Hospital Consumer Assessment of Healthcare Providers and Systems) and outcomes for myocardial infarction, heart failure, and pneumonia. The authors showed a significant correlation of the Yelp scores for five of six outcome measures, indicating that better ratings are associated with better medical outcomes. In addition, the study demonstrated a significant correlation of high ratings on Yelp and HCAHPS (*p* = 0.49; *p* < 0.001) as well as its domains (*p* ≤ 0.001 for all domains). Even though these results are valid for the hospital sector they demonstrate that online ratings in general may be more useful than is often thought [18].

We found only one study which focused on the association between online physician ratings and measures of clinical quality and conventional measures of patient experience [16]. The data contained 16,952 ratings of family practices from NHS Choices. These were compared with the results of the mail-based National General Practice Patient Survey containing approximately 2.1 million responses. The clinical data encompassed seven measures. Here, the authors showed significant associations between online ratings and the mail-based patient experience survey for all five assessed questions (*p* = 0.36 – 0.48, *p* < 0.001 for all) but only weak associations with measures of clinical care (Spearman p less than ±0.18, *p* < 0.001 for six of seven variables). Significant associations were shown for measures such as the proportion of patients with diabetes receiving flu vaccinations, controlled HbA1C in patients with diabetes, cervical screening rates, and admission rates for ambulatory care conditions.

These findings are partly in line with our presented results. We also demonstrated a strong association between online and conventional patient satisfaction survey results for both German PRWs. There again, regarding preventative services, results from the UK indicate a weak but significant association for cervical screening rates and for diabetic patients receiving flu vaccinations. Our results indicate an association for only one of ten measures with the preventative measure. Nevertheless, differences might be owed to the different measures used in the studies since we assessed the general preventative examination for patients aged 35 years or older. A similar conclusion can be drawn when we compare the results for the clinical indicators addressing the three chronic conditions diabetes type 2, coronary heart disease, and asthma. In contrast to the UK results, we found strong associations for two diabetes measures and one asthma measure. There again, no associations were found for the four coronary heart disease measures. One possible explanation for this result might be the age of patients since the literature has shown declining Internet [33–36] and PRW [10, 37] use with increasing age. The fact that the ages of patients in our study with coronary heart disease (72.83 years, SD 3.86) and diabetes (69.22 years, SD 3.31) were relatively high compared with the age of those with asthma (58.37 years, SD 10.33) might thus explain, at least to some extent, why none of the coronary heart disease measures was associated with the online ratings.

Regarding the cost-targeting measures, both studies detected meaningful associations. In the UK study, a very weak negative association was determined between ratings and low-cost statin prescriptions [16]. In contrast, we showed a strong association between the online ratings and the medication cost per case for three of four measures (*p* = 0.297 – 0.384, *p* < 0.05 for all) indicating that higher costs were related to better ratings. We further differentiated between general practitioners and specialists and determined the association to be true only for specialists and not for general practitioners (data not shown here). This finding might be explained to some extent by the long-term relationship of general practitioners with their patients [38] and the fact that specialists are consulted for more specialized interventions or because patients are suffering from more serious diseases [8, 14]. Patients might thus have a greater desire for getting medication prescribed when seeing a specialist.

In another study analyzing 386,000 national ratings from the US PRW RateMDs, Gao and colleagues investigated the association of online ratings with structural measures [2]. The authors found that online ratings were more positive for physicians who graduated in more recent years, are board-certified, graduated from highly rated medical schools, and those without malpractice claims. This might suggest a positive correlation between the online ratings and the physician quality, even though the magnitude was shown to be small. Our study can partly confirm those findings since a significant association between the age of the physician and the online ratings was found on one PRW. More recently, another study measured the association between online ratings from eight US PRWs and traditional quality measures of clinical and patient experience for a sample of 1299 physicians who completed an American Board of Internal Medicine Hypertension or Diabetes Practice Improvement Module [39]. In line with the results shown above, the authors also found small and statistically insignificant associations between online ratings and clinical quality measures as well as small but statistically significant associations with patient experience measures.

The results of this study extend the knowledge of previous studies since the patient per doctor ratio in a practice was strongly associated with all 10 included measures; i.e., the more patients physicians treat in a practice, the lower the ratings. This finding is not surprising but it highlights the importance of good physician-patient communication. Physicians should plan to spend sufficient time with patients rather than treating as many patients as possible. Of course, it is questionable whether physicians can dedicate more time to each patient in practice since most reimbursement systems do not include financial incentives for “talking medicine” treatment [40]. We further could not detect any significant correlations with clinical care measures for the elderly (e.g., medication therapy). This might demonstrate a limited usefulness of online ratings for older patients. However, this should be assessed more in detail in further studies.

## Conclusions

A recommendation as to whether or not patients should rely on online ratings to select a physician can be made in part. Patients who mainly focus on the satisfaction of other patients with a physician might select a physician on the basis of online ratings. Even though online ratings are likely to be biased [16], they are strongly associated with results from conventional patient surveys. Furthermore, patients who value a lower patient per doctor ratio might use the online ratings for choosing a physician. Whether or not patients can really expect consultations of longer duration should be addressed in future studies. Whether patients interested in the clinical quality of care of a physician should rely on online ratings to make a choice cannot be answered yet. Even though our results indicate strong associations with some types of diabetes and asthma, there is insufficient evidence to draw any strong conclusions. Consequently, it remains uncertain whether and to what extent online ratings reflect the quality of care [16]. The usefulness of online ratings especially for the elderly seems to be limited and should be addressed more in detail in the future.

### Limitations

There are some limitations that have to be taken into account when the results of this study are interpreted. First, our study adopted a cross-sectional design, so we were able to identify associations between exposure and outcomes but could not infer cause and effect. Second, the limited number of practices included in our study might have weakened our findings. However, since there are only limited data either on a nationwide basis or publicly available on the German healthcare system, we conducted this first study with a provider network which could report information on a large number of quality measures even though the number of practices was limited. Otherwise, we would have been able to include only a very limited number of quality measures. Third, because our study population is a convenience sample, it is not possible to generalize the results directly to the entire German physician population. Even though the median age of our study population (study sample: 53.40 years vs. German physician population: 53.30 years [41]) as well as the number of physicians per practice (study sample: 1.8 vs. German physician population: 1.5 [42]) is similar, the percentage of general practitioner is higher compared with the German physician population (49.23 % vs. 43.05 % [41]). But even more important might be that integrated health care networks often implement additional educational training, put a stronger emphasis on quality circle work, have selective contracts with health sickness funds (what might include different payment systems) etc. Fourth, our systematic search procedure was limited to the Medline database (via PubMed). We did not include further databases since it was not our primary aim to carry out a comprehensive and systematic literature review but to capture the literature in the most relevant database. However, we checked all references in the studies and also searched Google. Fifth, we were not able to include all German PRWs. Thus, our findings cannot be generalized for online ratings on other rating websites. Nevertheless, both websites play a major role in the German PRW movement [43].

## Additional file

Additional file 1:
**“The association between online ratings and structural and quality_Bivariate analysis”, PDF format.** (PDF 534 kb)

## References

[1] Emmert M, Sander U, Esslinger AS, Maryschok M, Schoffski O (2012). Public reporting in Germany: the content of physician rating websites. Methods Inf Med.

[2] Gao GG, McCullough JS, Agarwal R, Jha AK (2012). A changing landscape of physician quality reporting: analysis of patients’ online ratings of their physicians over a 5-year period. J Med Internet Res.

[3] López A, Detz A, Ratanawongsa N, Sarkar U (2012). What patients Say about their doctors online: a qualitative content analysis. J Gen Intern Med.

[4] Emmert M, Meier F, Heider A, Dürr C, Sander U (2014). What do patients say about their physicians? An analysis of 3000 narrative comments posted on a German physician rating website. Health Policy.

[5] Bakhsh W, Mesfin A (2014). Online ratings of orthopedic surgeons: analysis of 2185 reviews. Am J Orthop.

[6] Reimann S, Strech D (2010). The representation of patient experience and satisfaction in physician rating sites. A criteria-based analysis of English- and German-language sites. BMC Health Serv Res.

[7] Greaves F, Millett C (2012). Consistently increasing numbers of online ratings of healthcare in England. J Med Internet Res.

[8] Emmert M, Meier F (2013). An analysis of online evaluations on a physician rating website: evidence from a German public reporting instrument. J Med Internet Res.

[9] Strech D, Reimann S (2012). Deutschsprachige arztbewertungsportale: Der status quo ihrer bewertungskriterien, bewertungstendenzen und nutzung. German language physician rating sites: the status Quo of evaluation criteria, evaluation tendencies and utilization. Gesundheitswesen.

[10] Emmert M, Meier F, Pisch F, Sander U (2013). Physician choice making and characteristics associated with using physician-rating websites: cross-sectional study. J Med Internet Res.

[11] Greaves F, Millett C, Nuki P (2014). England’s experience incorporating “anecdotal” reports from consumers into their national reporting system: lessons for the united states of what to Do or Not to Do?. Med Care Res Rev.

[12] Black EW, Thompson LA, Saliba H, Dawson K, Black NMP (2009). An analysis of healthcare providers’ online ratings. Inform Prim Care.

[13] Kadry B, Chu L, Kadry B, Gammas D, Macario A (2011). Analysis of 4999 online physician ratings indicates that most patients give physicians a favorable rating. J Med Internet Res.

[14] Lagu T, Hannon NS, Rothberg MB, Lindenauer PK (2010). Patients’ evaluations of health care providers in the era of social networking: an analysis of physician-rating websites. J Gen Intern Med.

[15] Sobin L, Goyal P (2014). Trends of online ratings of otolaryngologists: what do your patients really think of you?. JAMA Otolaryngol Head Neck Surg.

[16] Greaves F, Pape UJ, Lee H, Smith DM, Darzi A, Majeed A, Millett C (2012). Patients’ ratings of family physician practices on the internet: usage and associations with conventional measures of quality in the english national health service. J Med Internet Res.

[17] Mackay B (2007). RateMDs.com nets ire of Canadian physicians. Can Med Assoc J.

[18] Greaves F, Pape UJ, King D, Darzi A, Majeed A, Wachter RM, Millett C (2012). Associations between Web-based patient ratings and objective measures of hospital quality. Arch Intern Med.

[19] Integrated Health Care Network Quality and Efficiency (Ed.): *Quality Report 2012.* Nuremberg; 2013

[20] Integrated Health Care Network Quality and Efficiency (Ed.): *Patientenzeitschrift “Pumperlgsund in Nürnberg”.* Nuremberg; 2014.

[21] Holt S, Schmiedl S, Thürmann PA (2010). Potentially inappropriate medications in the elderly: the PRISCUS list. Dtsch Arztebl Int.

[22] Bertelsmann Stiftung (Ed.): *Weisse Liste - Fragen zur Arztsuche. Gütersloh*; 2014

[23] Bertelsmann Stiftung (Ed.): *Weisse Liste - Die Fragebögen: Haus- und Fachärzte. Gütersloh*; 2012

[24] Verhoef LM, Van De B, Tom H, Engelen Lucien JLPG, Schoonhoven L, Kool RB (2014). Social media and rating sites as tools to understanding quality of care: a scoping review. J Med Internet Res.

[25] Beattie M, Lauder W, Atherton I, Murphy DJ (2014). Instruments to measure patient experience of health care quality in hospitals: a systematic review protocol. Syst Rev.

[26] Rathert C, Wyrwich MD, Boren SA (2013). Patient-centered care and outcomes: a systematic review of the literature. Med Care Res Rev.

[27] Delpierre C, Cuzin L, Fillaux J, Alvarez M, Massip P, Lang T (2004). A systematic review of computer-based patient record systems and quality of care: more randomized clinical trials or a broader approach?. Int J Qual Health Care.

[28] Seddon ME, Marshall MN, Campbell SM, Roland MO (2001). Systematic review of studies of quality of clinical care in general practice in the UK, Australia and New Zealand. Qual Health Care.

[29] Derksen F, Bensing J, Lagro-Janssen A (2013). Effectiveness of empathy in general practice: a systematic review. Br J Gen Pract.

[30] Doyle C, Lennox L, Bell D (2013). A systematic review of evidence on the links between patient experience and clinical safety and effectiveness. BMJ Open.

[31] Greaves F, Pape UJ, King D, Darzi A, Majeed A, Wachter RM, Millett C (2012). Associations between Internet-based patient ratings and conventional surveys of patient experience in the English NHS: an observational study. BMJ Qual Saf.

[32] Bardach NS, Asteria-Peñaloza R, Boscardin WJ, Dudley RA (2013). The relationship between commercial website ratings and traditional hospital performance measures in the USA. BMJ Qual Saf.

[33] Fox S: *The social life of health information.* Pew Research Center (Ed.), Washington D.C.; 2011

[34] Office for National Statistics (Ed.): *Internet Access - Households and Individuals 2012.* London, UK; 2012

[35] Iverson SA, Howard KB, Penney BK (2008). Impact of internet use on health-related behaviors and the patient-physician relationship: a survey-based study and review. J Am Osteopath Assoc.

[36] Smith-Barbaro PA, Licciardone JC, Clarke HF, Coleridge ST (2001). Factors associated with intended use of a Web site among family practice patients. J Med Internet Res.

[37] Terlutter R, Bidmon S, Röttl J (2014). Who uses physician-rating websites? Differences in sociodemographic variables, psychographic variables, and health status of users and nonusers of physician-rating websites. J Med Internet Res.

[38] Donahue KE, Ashkin E, Pathman DE (2005). Length of patient-physician relationship and patients’ satisfaction and preventive service use in the rural south: a cross-sectional telephone study. BMC Fam Pract.

[39] Gray BM, Vandergrift JL, Gao GG, McCullough JS, Lipner RS: Website ratings of physicians and their quality of care. *JAMA Intern Med*. 2014, Dec 1. [Epub ahead of print] 10.1001/jamainternmed.2014.6291 25437252

[40] Koch K, Gehrmann U, Sawicki PT (2007). Primärärztliche Versorgung in Deutschland im internationalen Vergleich. Ergebnisse einer strukturvalidierten Ärztebefragung. Deutsches Ärzteblatt.

[41] National Association of Statutory Health Insurance Physicians (Ed.): *Statistische Informationen aus dem Bundesarztregister, Bundesgebiet insgesamt (Stand: 31.12.2013)*. Berlin, Germany; 2014.

[42] Information System of the Federal Health Monitoring (Ed.): *Staff in medical practices and personnel expenditure. Classification (2007): years, Germany, staff/expenditure, revenue groups, type of practice*. Bonn, Germany. 2009.

[43] Emmert M, Meszmer N, Schöffski O: Arztbewertungsportale im Internet: Eine aktuelle Bestandsaufnahme. IMPLICONplus – Gesundheitspolitische Analysen 2014. Manfred Albring (Ed.), Berlin; 2014

