# Supplementary material for: A cross-sectional study assessing the association between online ratings and structural and quality of care measures: results from two German physician rating websites
Source: BMC Health Serv Res. 2015 Sep 24;15:414. doi: 10.1186/s12913-015-1051-5 (PMC4582723; doi:10.1186/s12913-015-1051-5)
Supplement: Additional file 1: — “The association between online ratings and structural and quality_Bivariate analysis”, PDF format. (PDF 534 kb) [file 12913_2015_1051_MOESM1_ESM.pdf]

Bivariate analysis - Weisse Liste

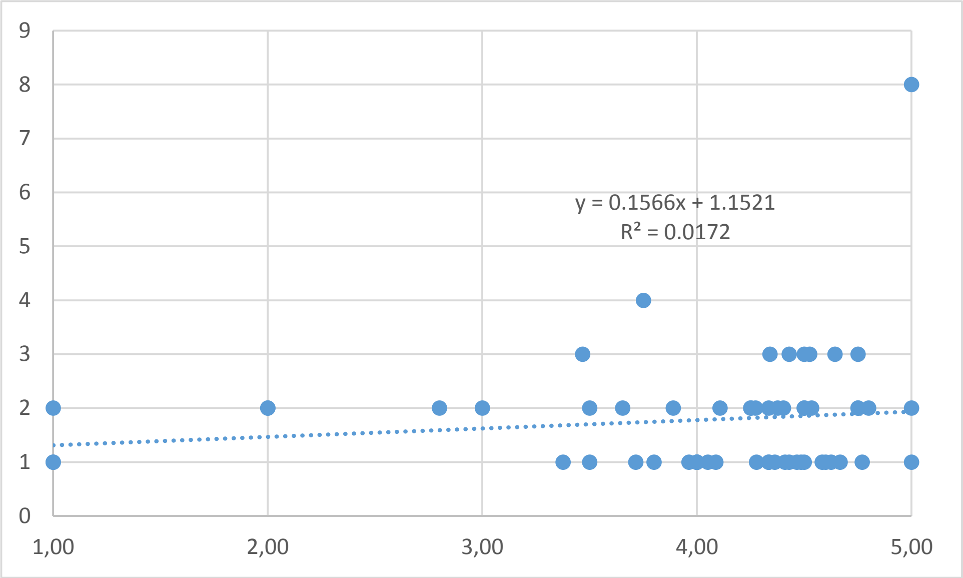

Figure 1: Number of physicians per practice and online rating Weisse Liste (Indicator 1)

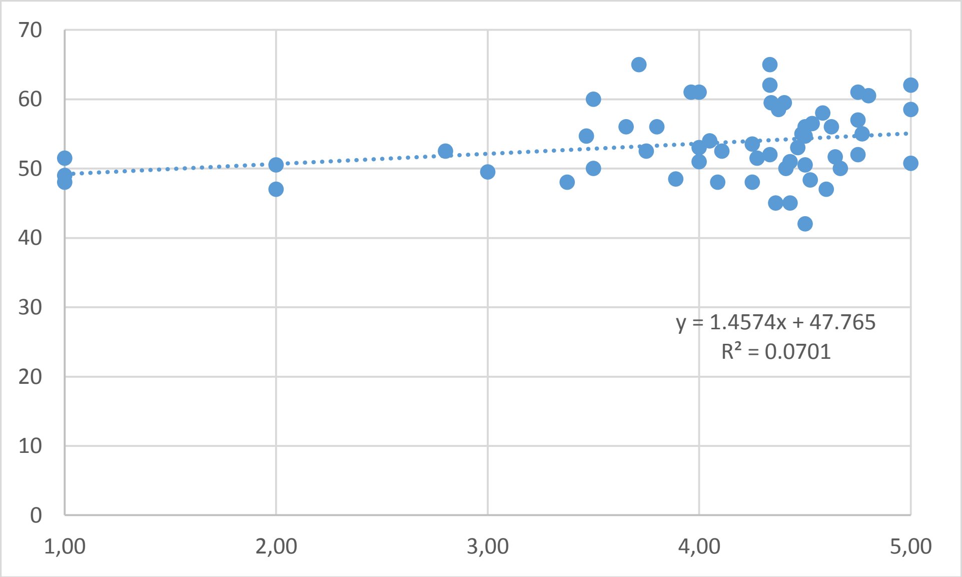

Figure 2: Age of the physicians per practice (average) and online rating Weisse Liste (Indicator 2)

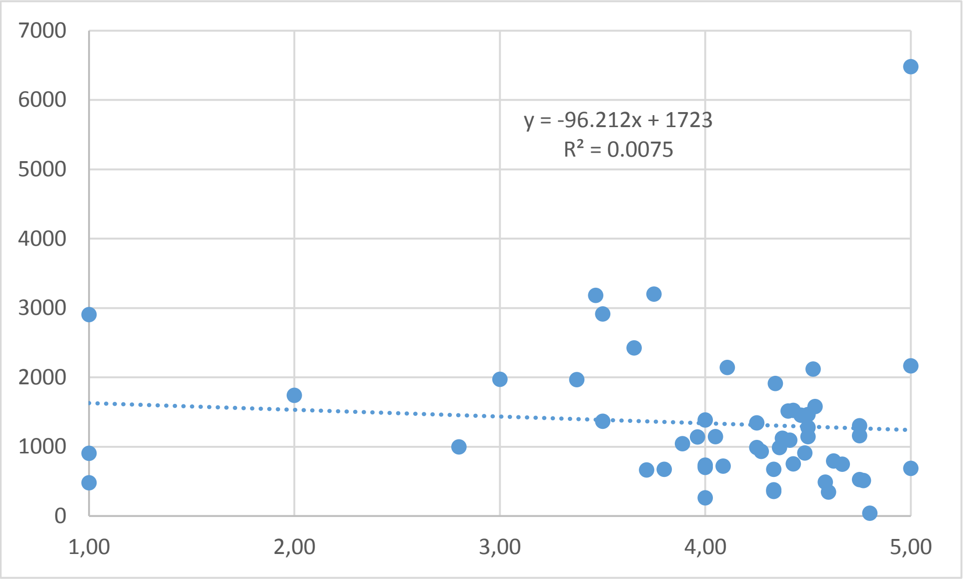

Figure 3: Patients per practice per quarter (average) and online rating Weisse Liste (Indicator 3)

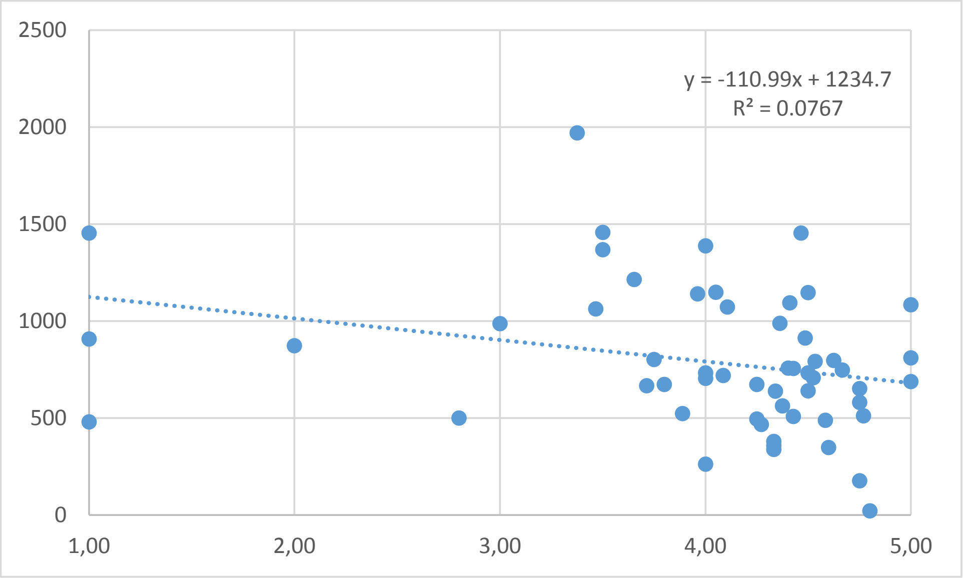

Figure 4: Patients per doctor ratio and online rating Weisse Liste (Indicator 4)

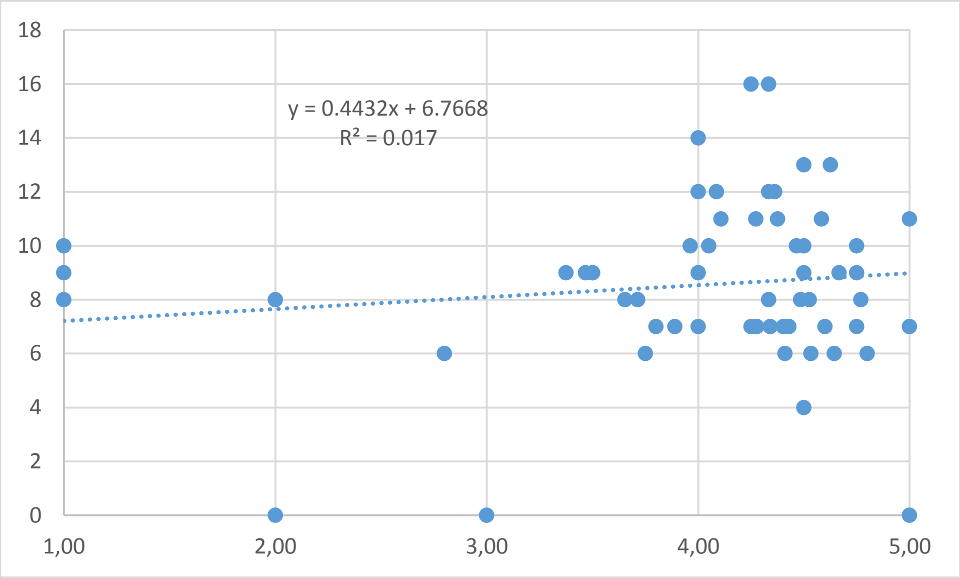

Figure 5: Quality circle visits (values practice-related) and online rating Weisse Liste (Indicator 5)

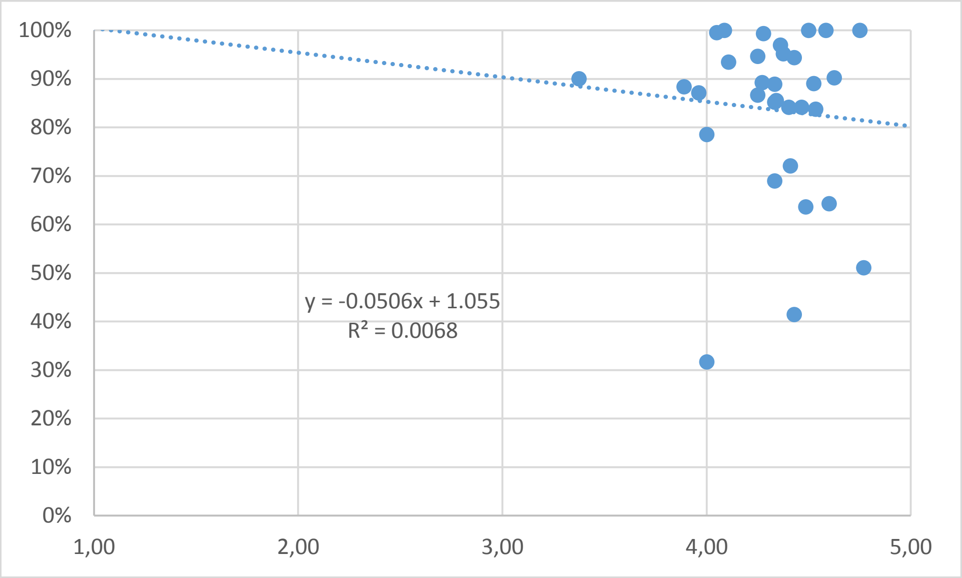

Figure 6: Chronically ill QuE-patients (Q4/2012) (in percentage) and online rating Weisse Liste (Indicator 6)

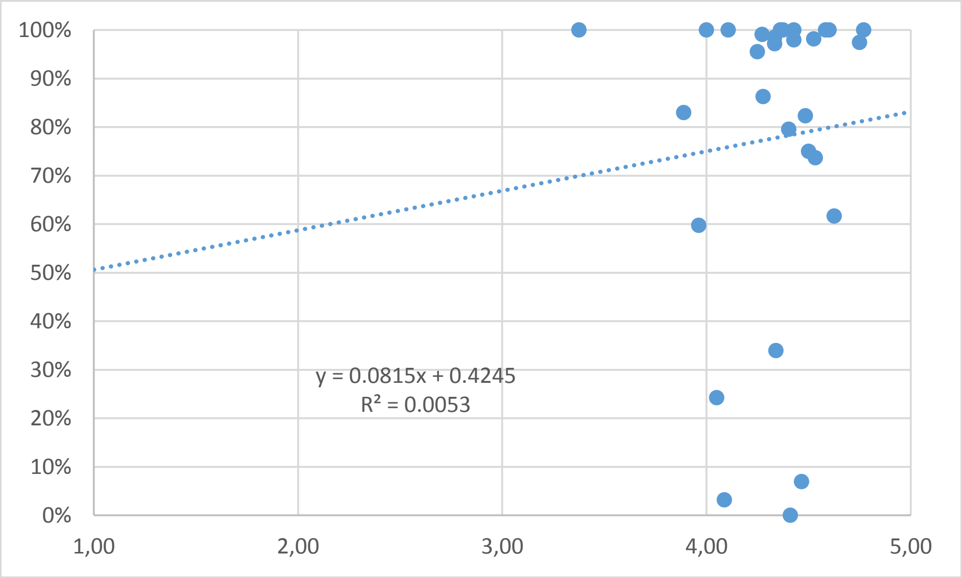

Figure 7: Patients with a diabetic retinal exam within the last 12 months (in percentage) and online rating Weisse Liste (Indicator 7)

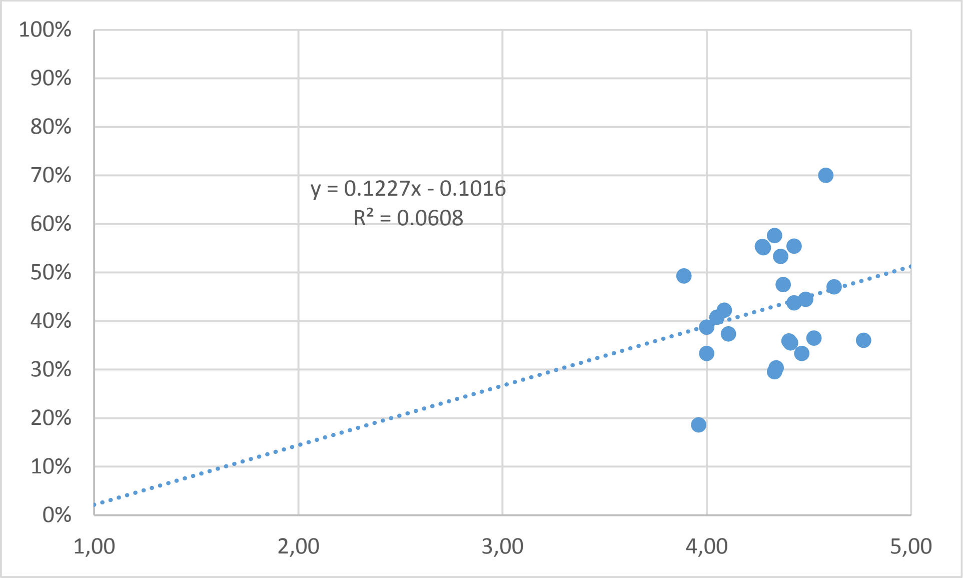

Figure 8: Patients who had an ophthalmological examination in 2012 and online rating Weisse Liste (Indicator 8)

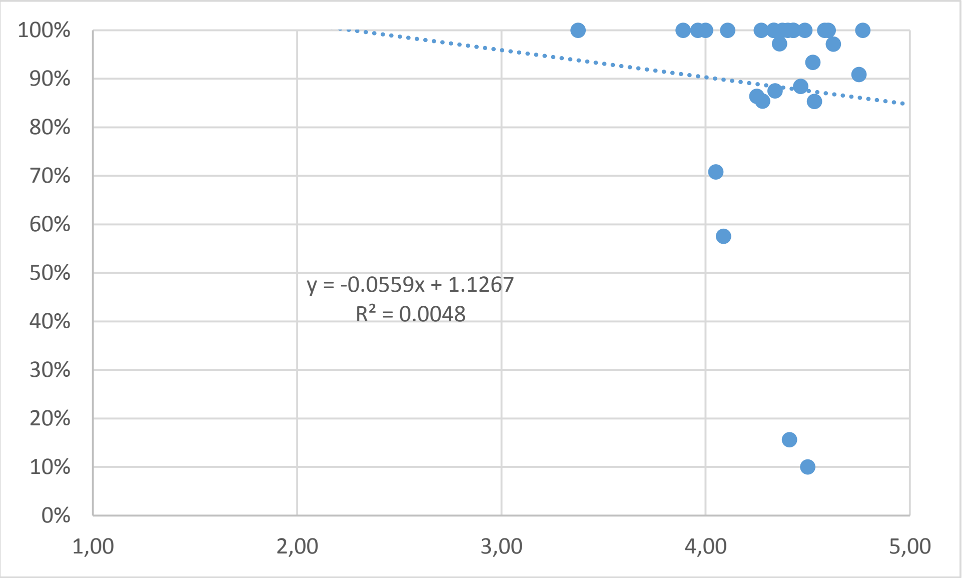

Figure 9: Patients who have been prescribed antiplatelet agents (in percentage) and online rating Weisse Liste (Indicator 9)

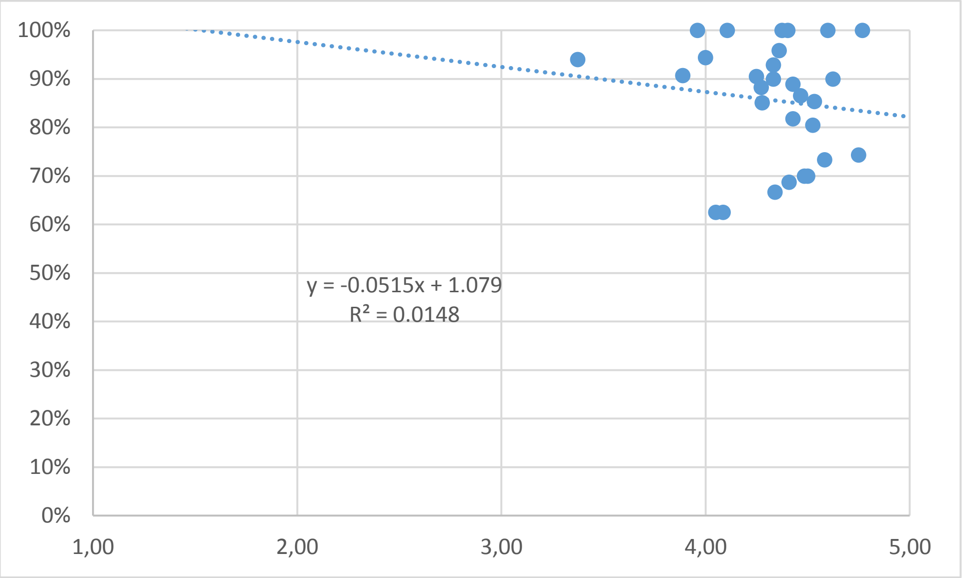

Figure 10: Patients who have been prescribed beta-blockers (in percentage) and online rating Weisse Liste (Indicator 10)

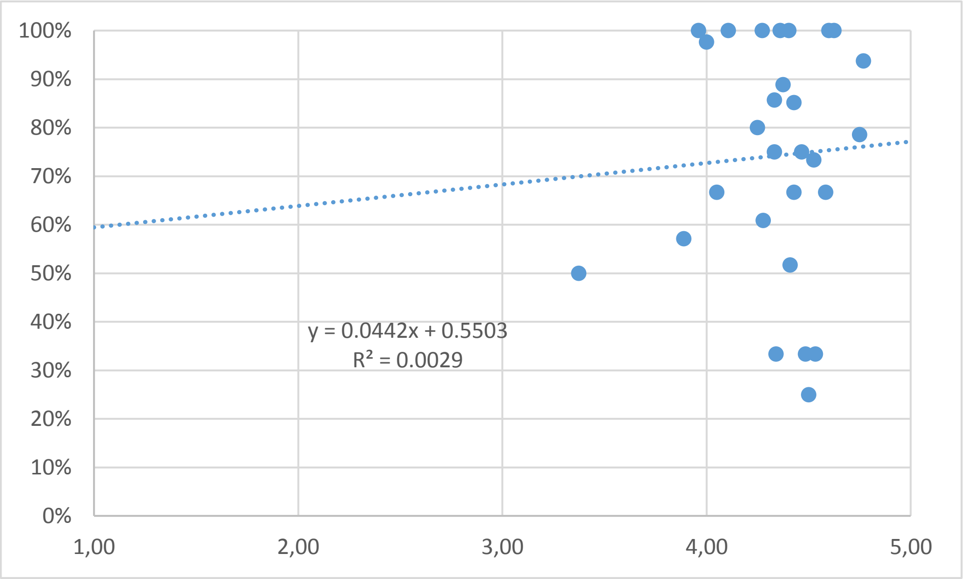

Figure 11: Patients with cardiac insufficiency who have been prescribed ACE inhibitors (in percentage) and online rating Weisse Liste (Indicator 11)

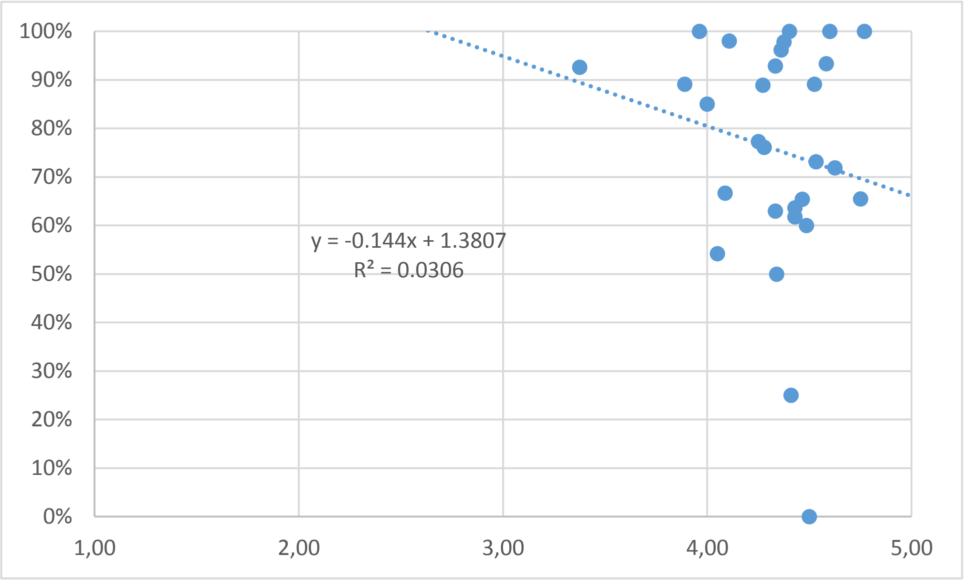

Figure 12: Patients who have been prescribed CHD statins (in percentage) and online rating Weisse Liste (Indicator 12)

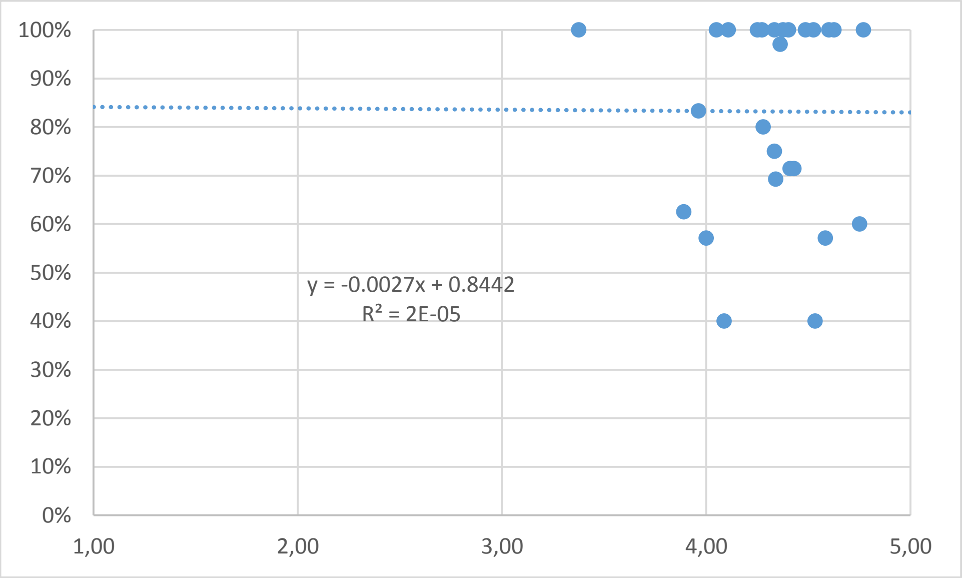

Figure 13: Patients with long-term medication who have been prescribed inhaled corticosteroids (in percentage) and online rating Weisse Liste (Indicator 13)

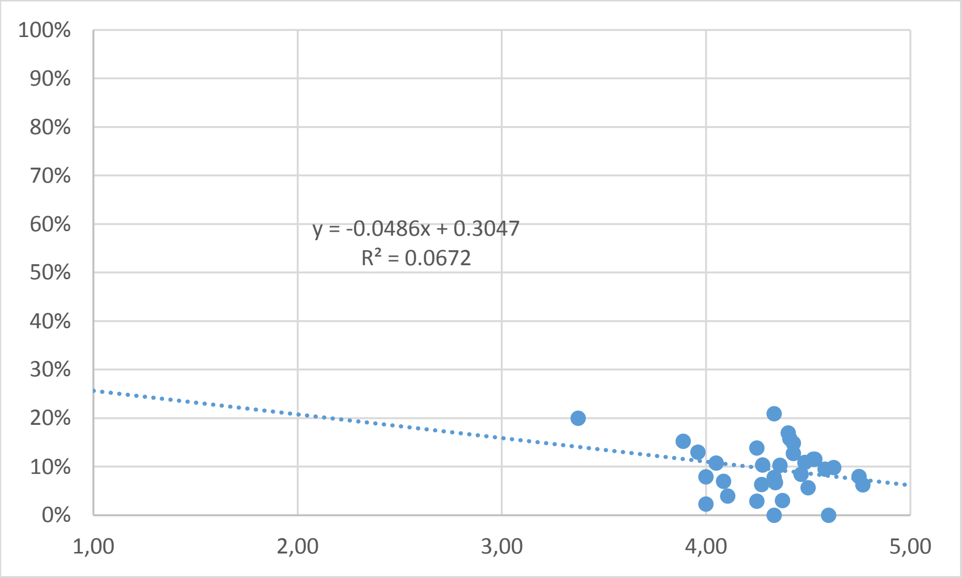

Figure 14: Polypharmacy: Patients aged 65 years or older with more than eight prescribed medications (Q4/2012) (in percentage) and online rating Weisse Liste (Indicator 14)

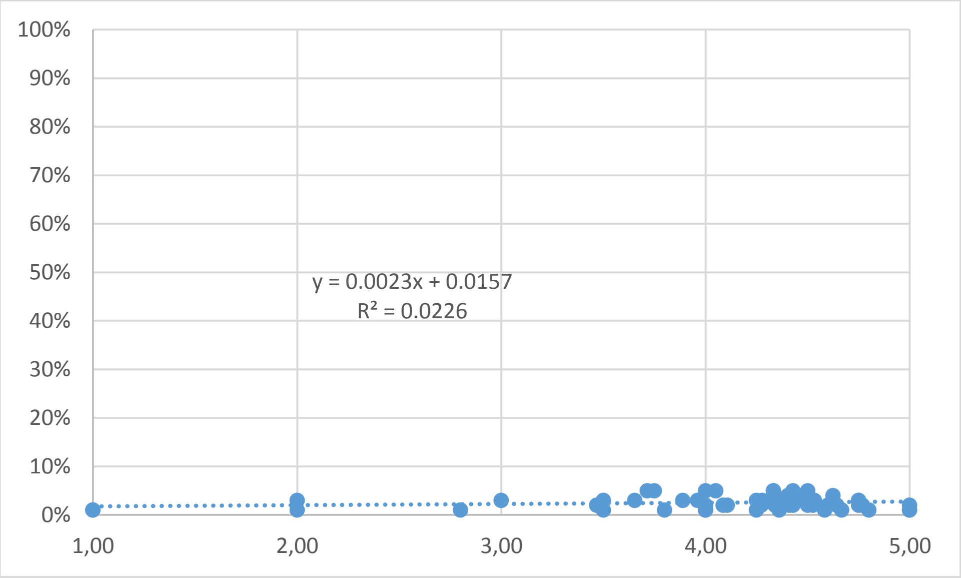

Figure 15: PRISCUS medication (2011) (in percentage) and online rating Weisse Liste (Indicator 15)

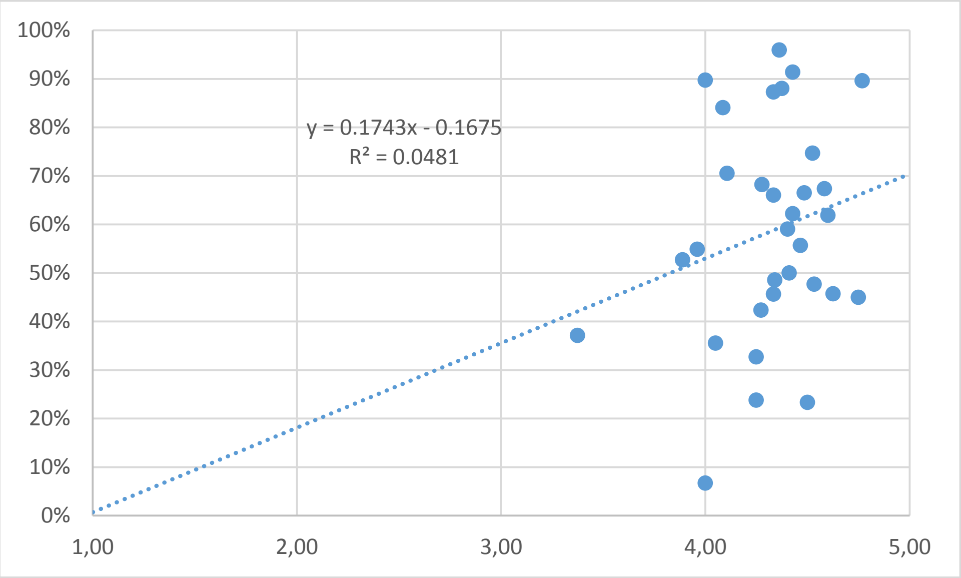

Figure 16: Patients aged 35 or older with a general preventive examination (in percentage) and online rating Weisse Liste (Indicator 16)

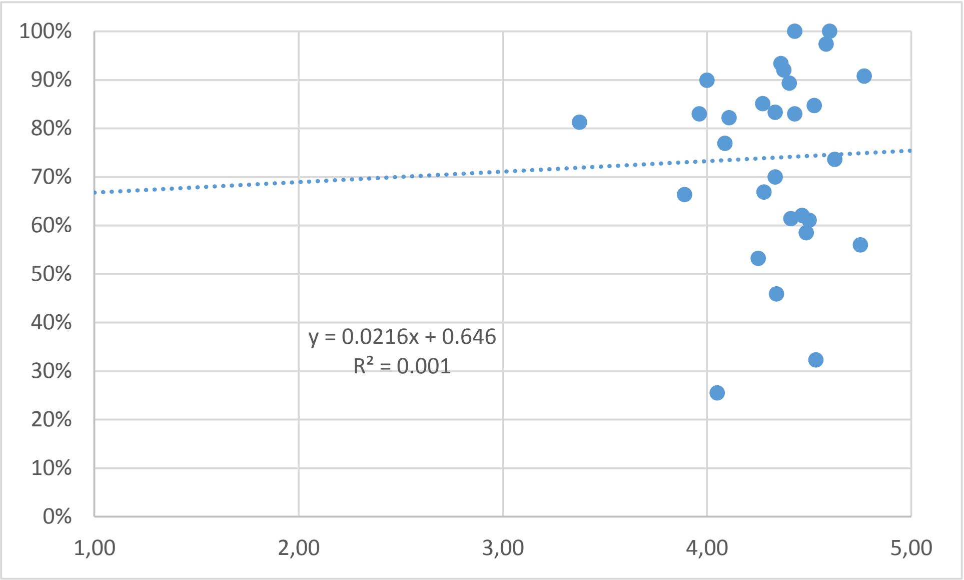

Figure 17: Patients who reached individual HbA1c-target values (in percentage) and online rating Weisse Liste (Indicator 17)

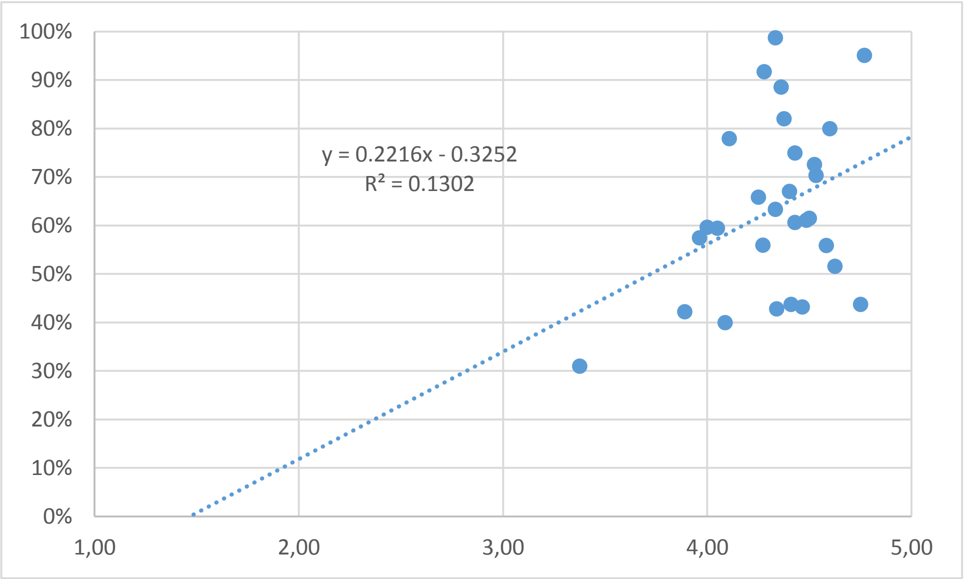

Figure 18: Patients with hypertension who show a normotensive blood pressure (in percentage) and online rating Weisse Liste (Indicator 18)

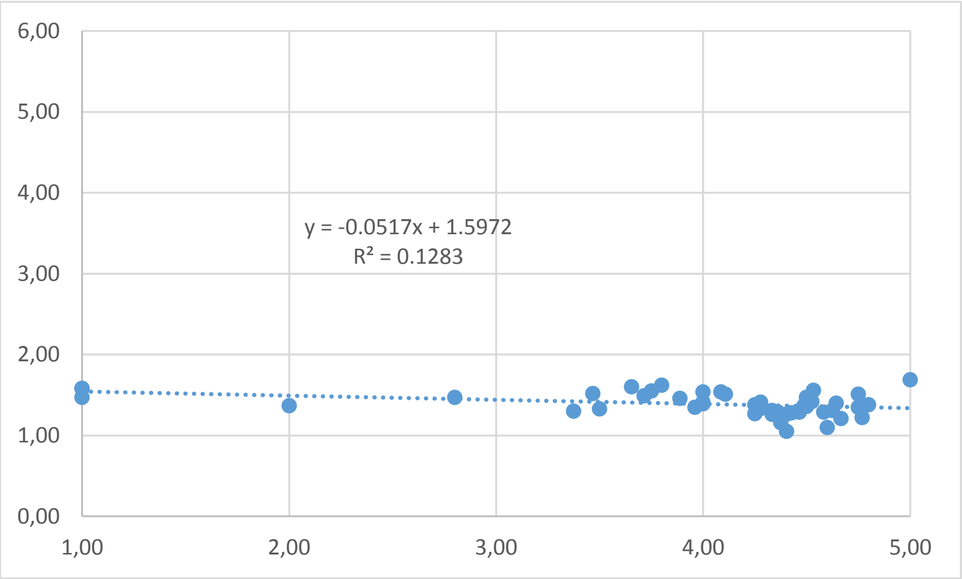

Figure 19: Offline patient survey 2012 (practice-related) and online rating Weisse Liste (Indicator 19)

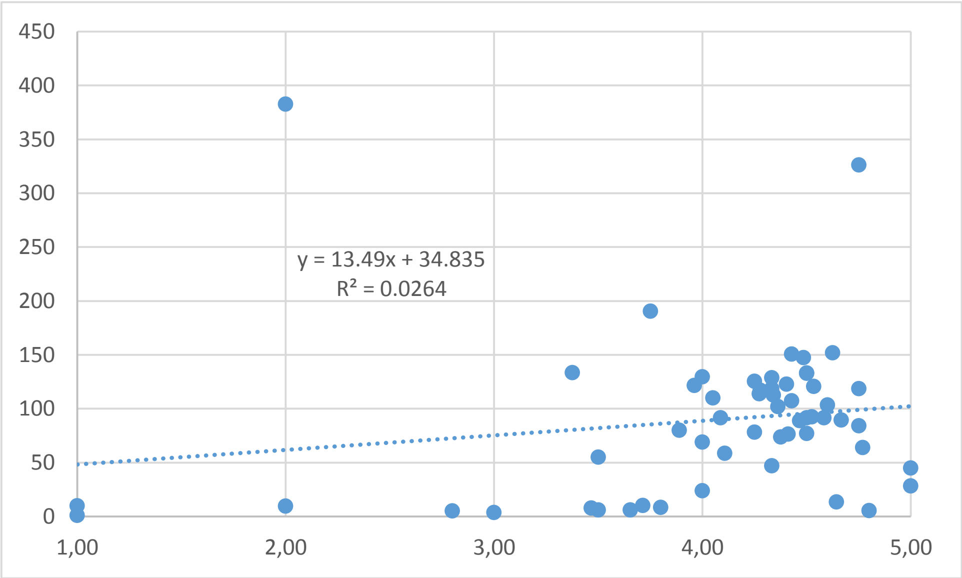

Figure 20: Cost per case (average 2012) and online rating Weisse Liste (Indicator 20)

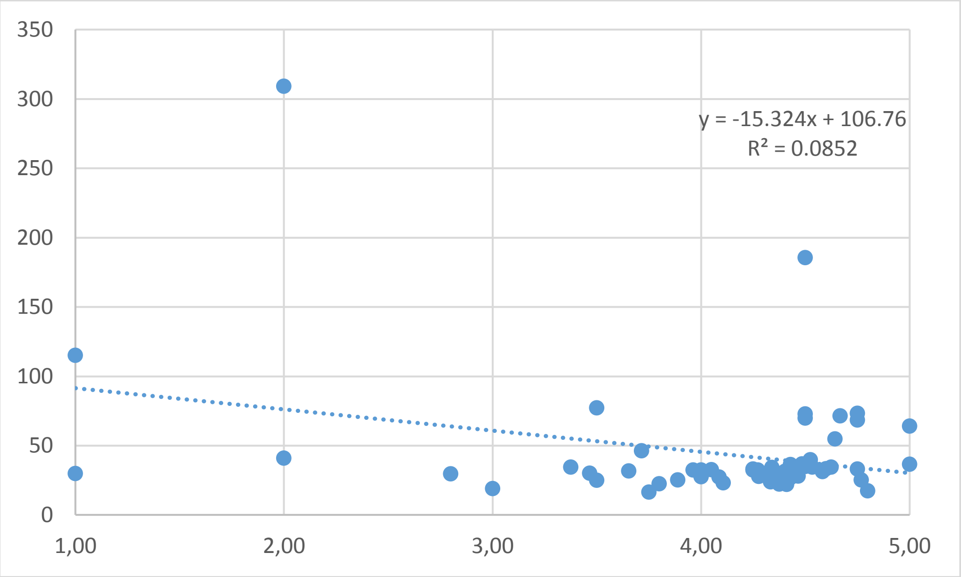

Figure 21: Cost per prescription (average 2012) and online rating Weisse Liste (Indicator 21)

Bivariate analysis – jameda

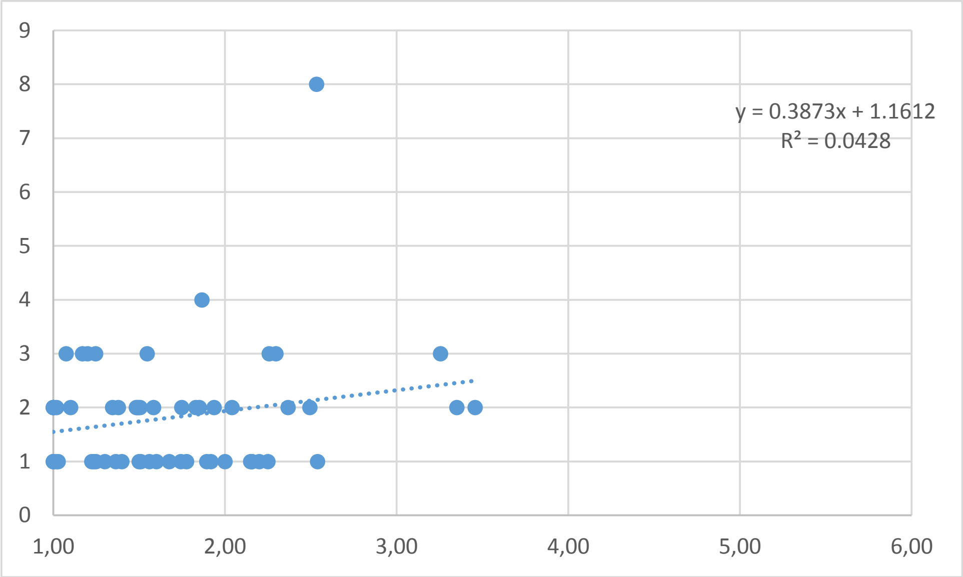

Figure 22: Number of physicians per practice and online rating jameda (Indicator 1)

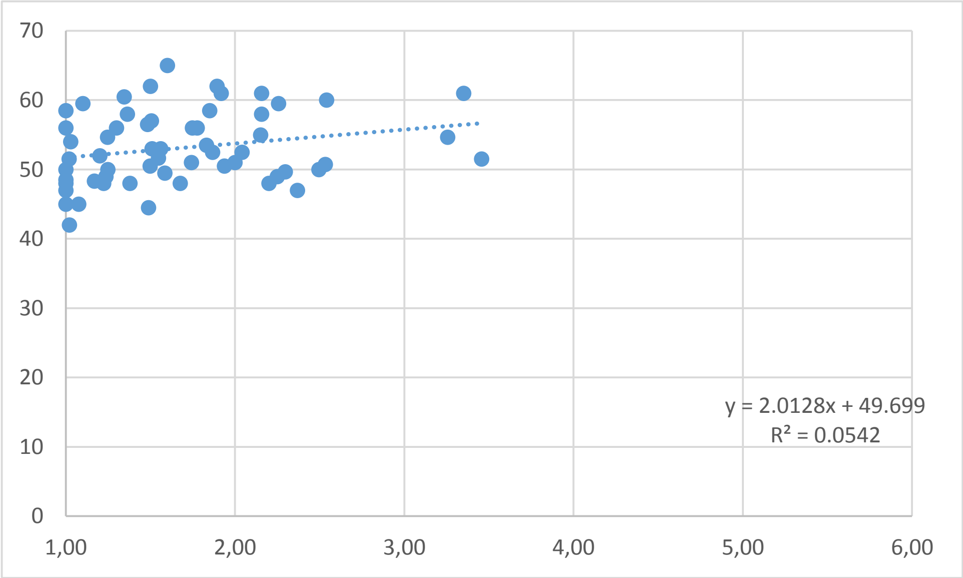

Figure 23: Age of the physicians per practice (average) and online rating jameda (Indicator 2)

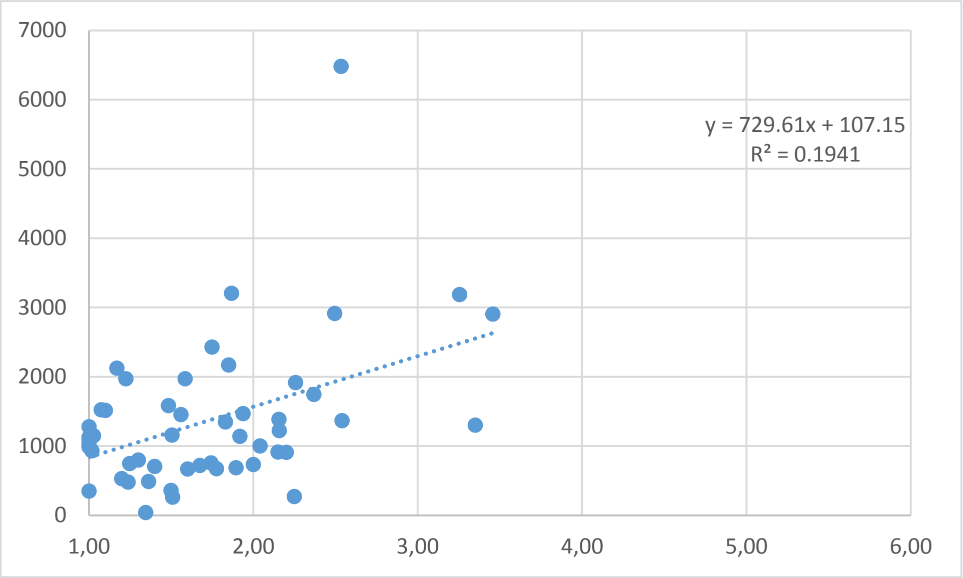

Figure 24: Patients per practice per quarter (average) and online rating jameda (Indicator 3)

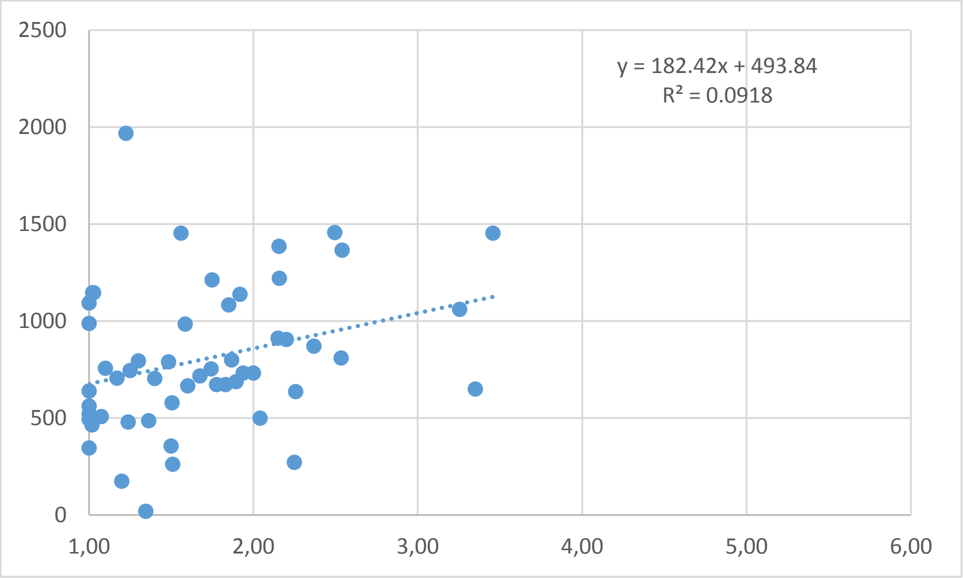

Figure 25: Patients per doctor ratio and online rating jameda (Indicator 4)

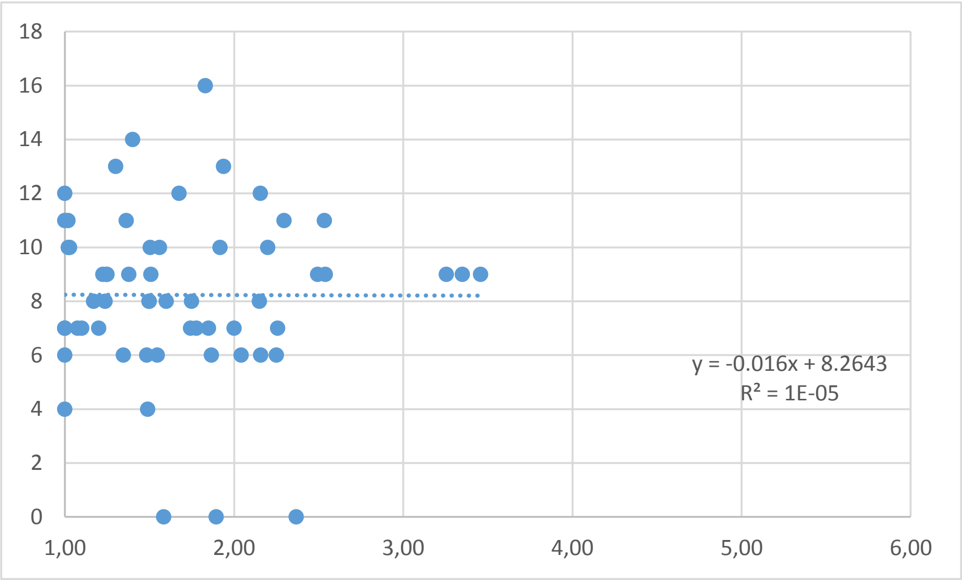

Figure 26: Quality circle visits (values practice-related) and online rating jameda (Indicator 5)

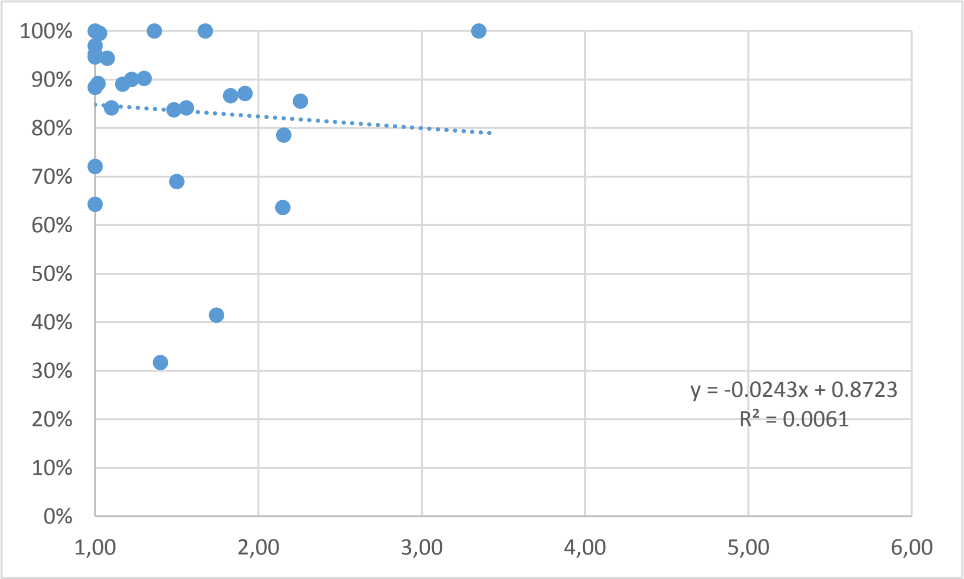

Figure 27: Chronically ill QuE-patients (Q4/2012) (in percentage) and online rating jameda (Indicator 6)

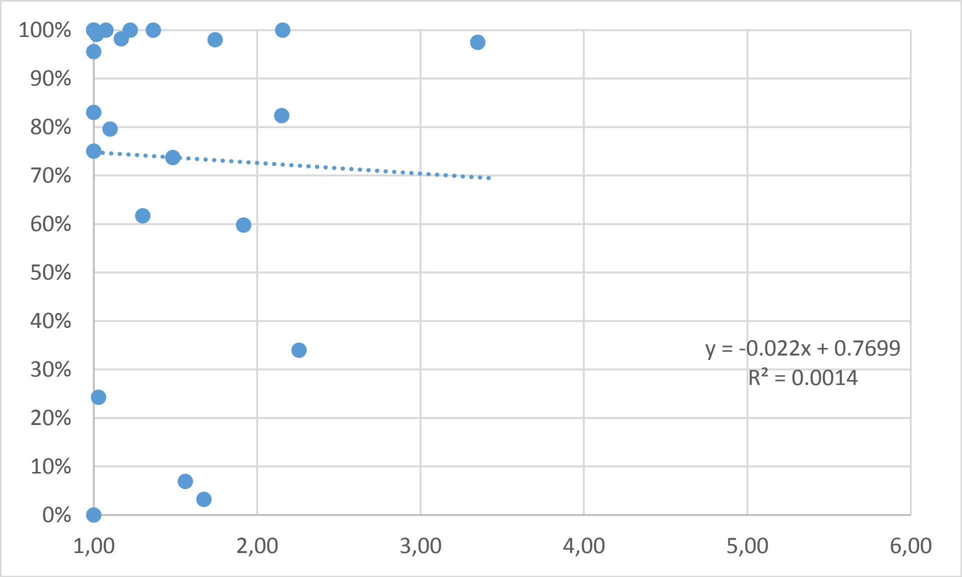

Figure 28: Patients with a diabetic retinal exam within the last 12 months (in percentage) and online rating jameda (Indicator 7)

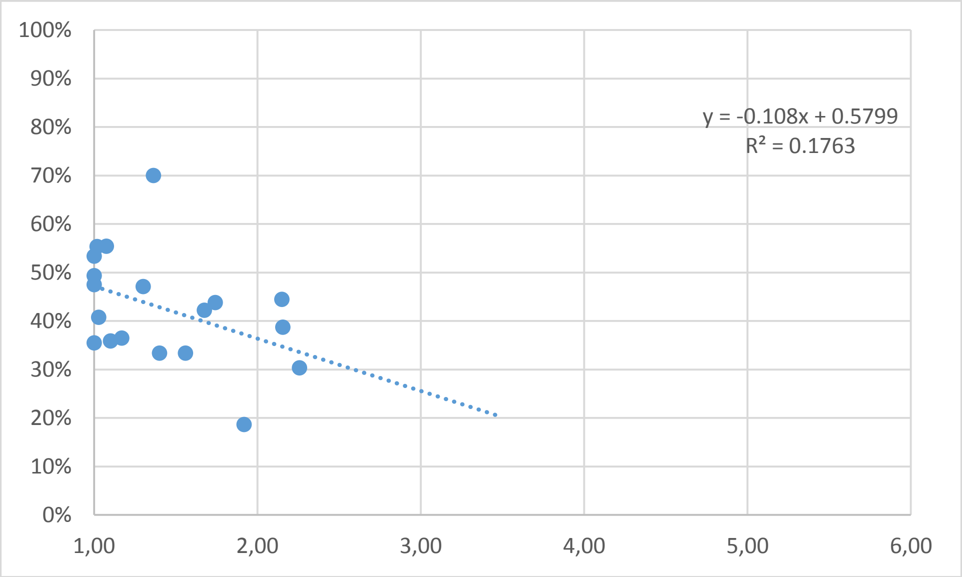

Figure 29: Patients who had an ophthalmological examination in 2012 and online rating jameda (Indicator 8)

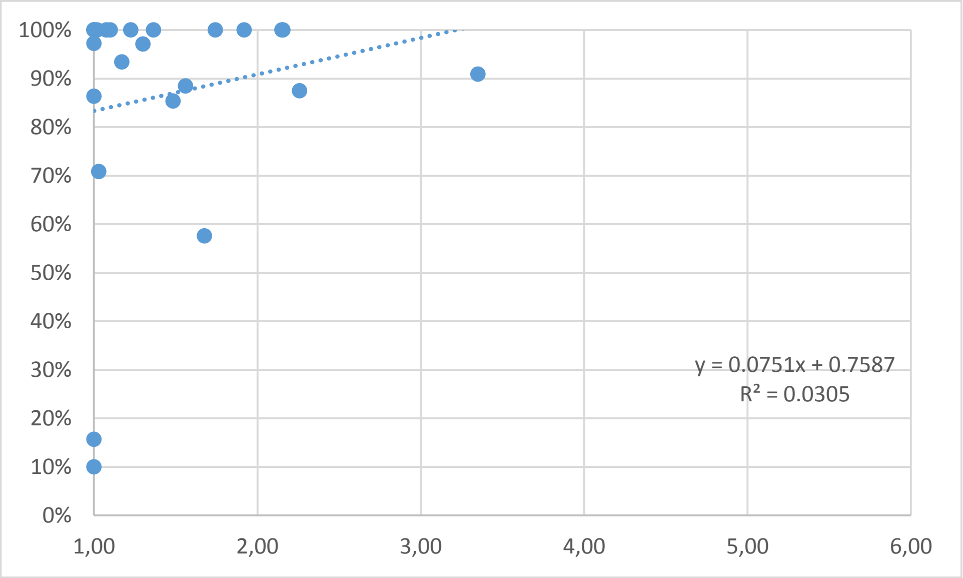

Figure 30: Patients who have been prescribed antiplatelet agents (in percentage) and online rating jameda (Indicator 9)

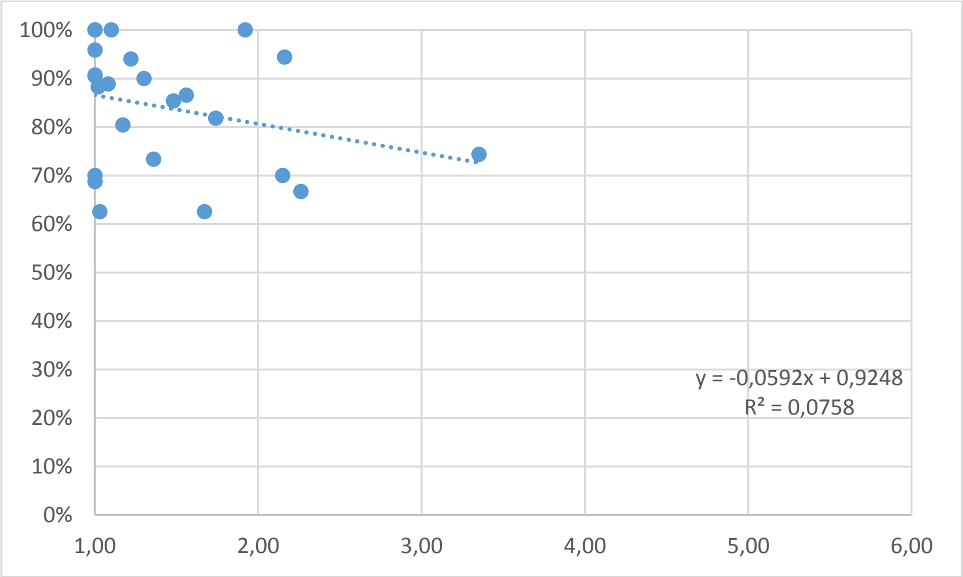

Figure 31: Patients who have been prescribed beta-blockers (in percentage) and online rating jameda (Indicator 10)

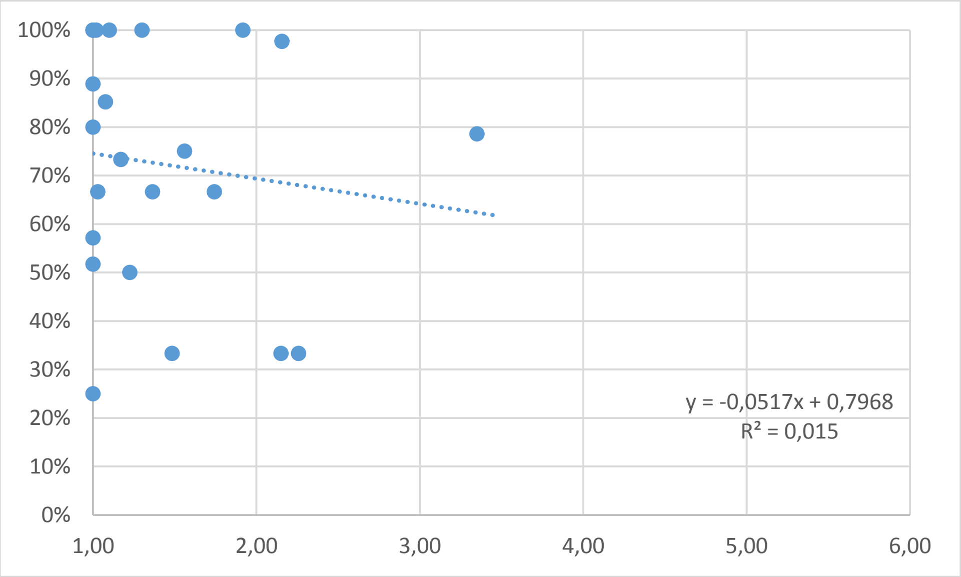

Figure 32: Patients with cardiac insufficiency who have been prescribed ACE inhibitors (in percentage) and online rating jameda (Indicator 11)

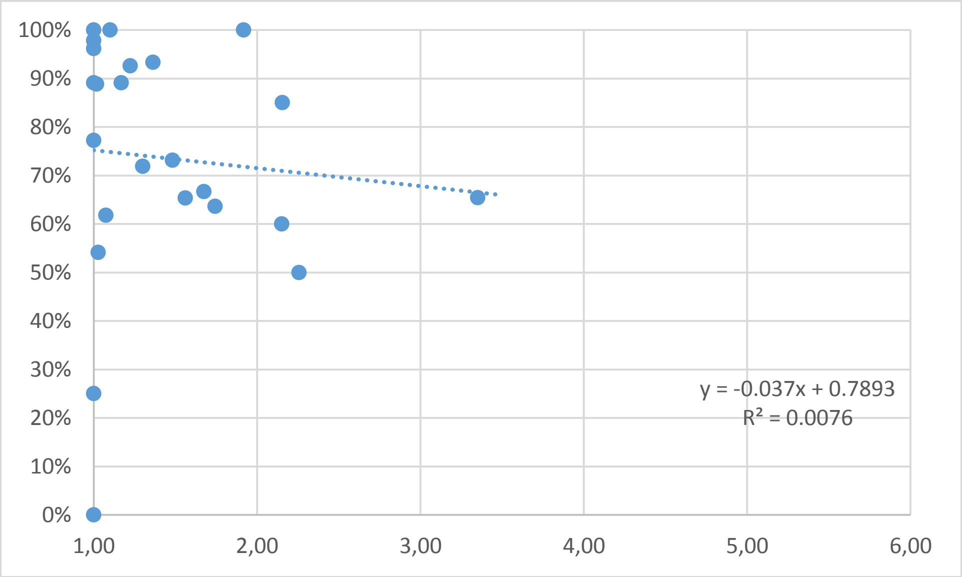

Figure 33: Patients who have been prescribed CHD statins (in percentage) and online rating jameda (Indicator 12)

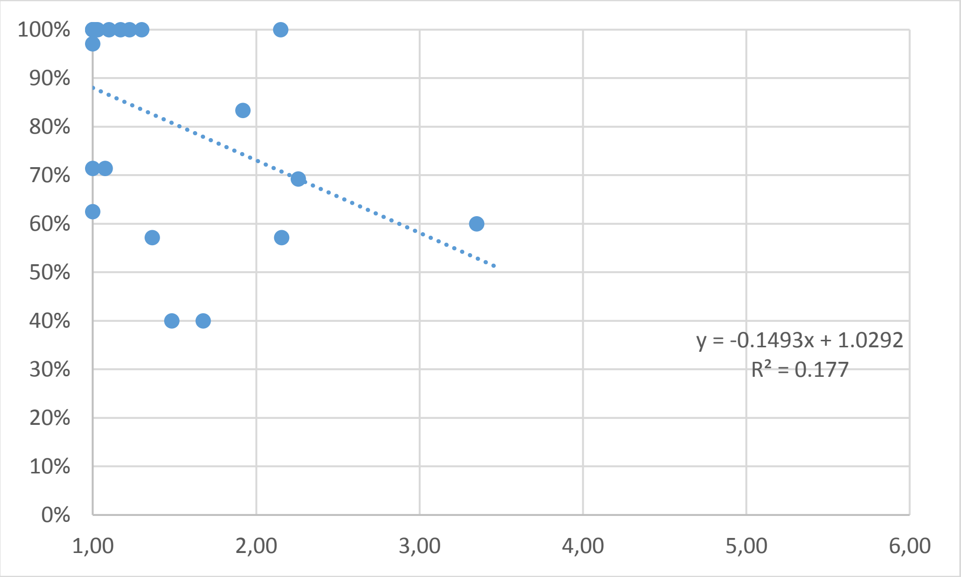

Figure 34: Patients with long-term medication who have been prescribed inhaled corticosteroids (in percentage) and online rating jameda (Indicator 13)

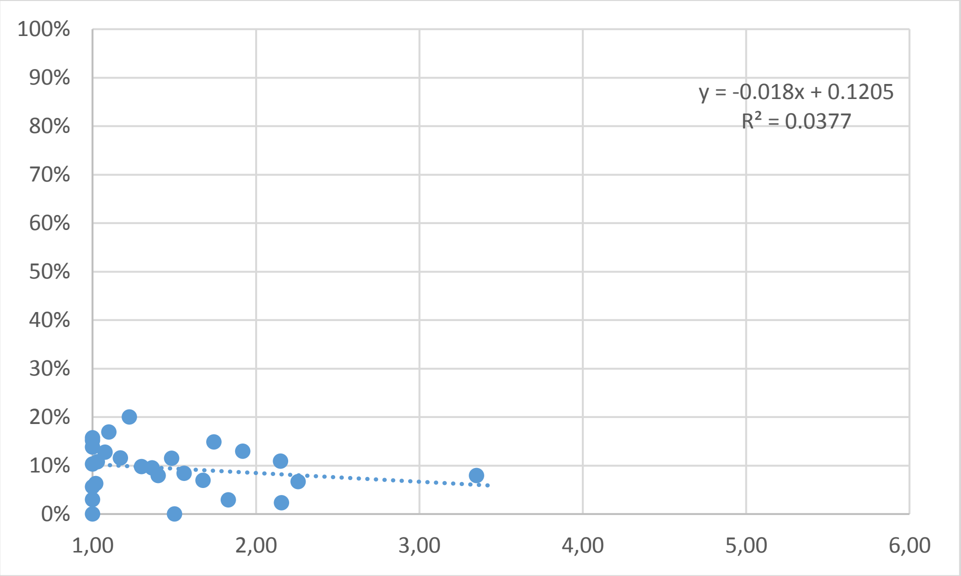

Figure 35: Polypharmacy: Patients aged 65 years or older with more than eight prescribed medications (Q4/2012) (in percentage) and online rating jameda (Indicator 14)

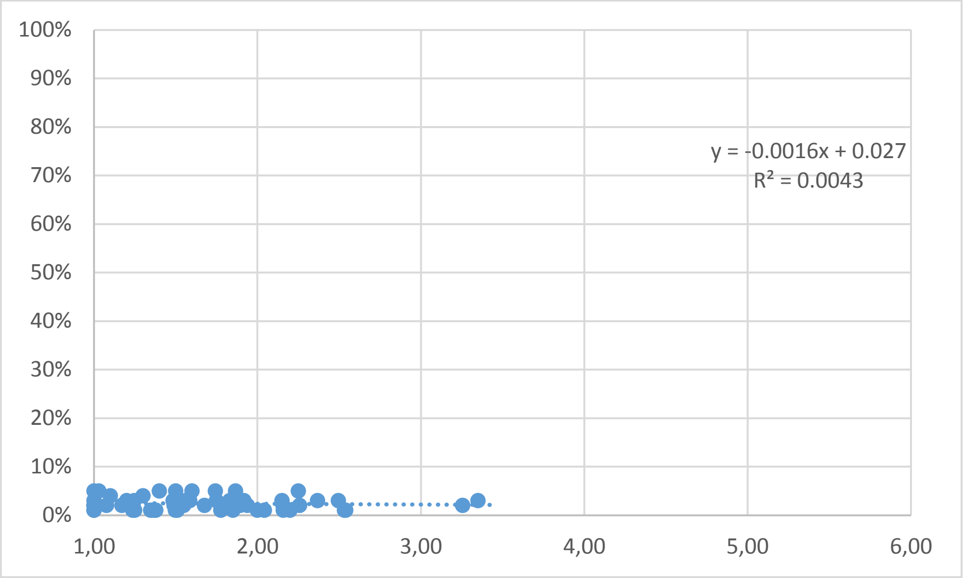

Figure 36: PRISCUS medication (2011) (in percentage) and online rating jameda (Indicator 15)

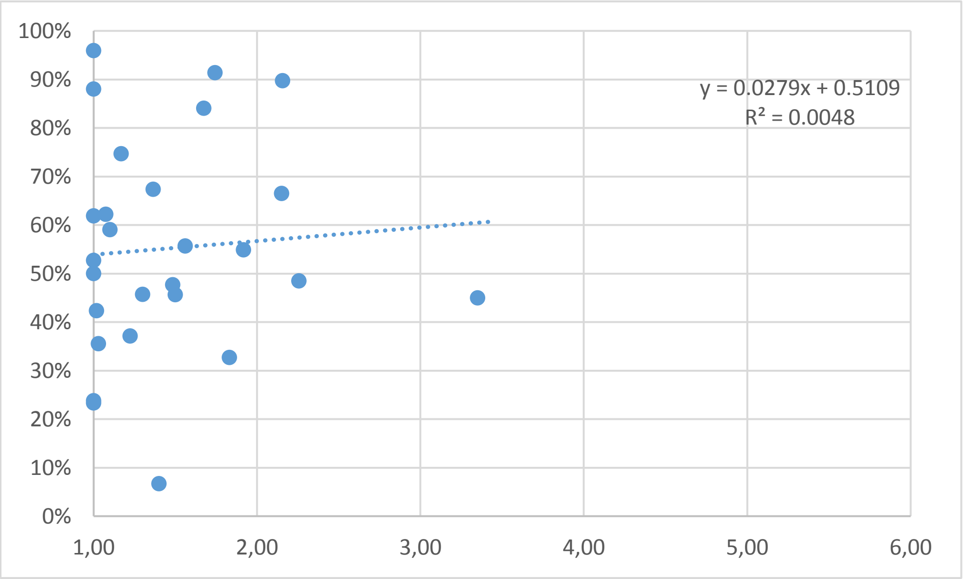

Figure 37: Patients aged 35 or older with a general preventive examination (in percentage) and online rating jameda (Indicator 16)

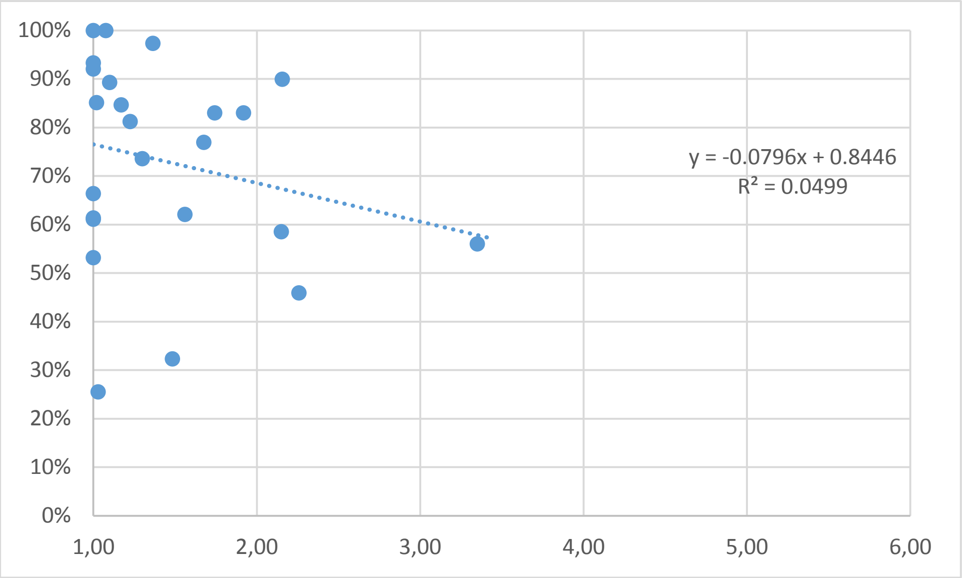

Figure 38: Patients who reached individual HbA1c-target values (in percentage) and online rating jameda (Indicator 17)

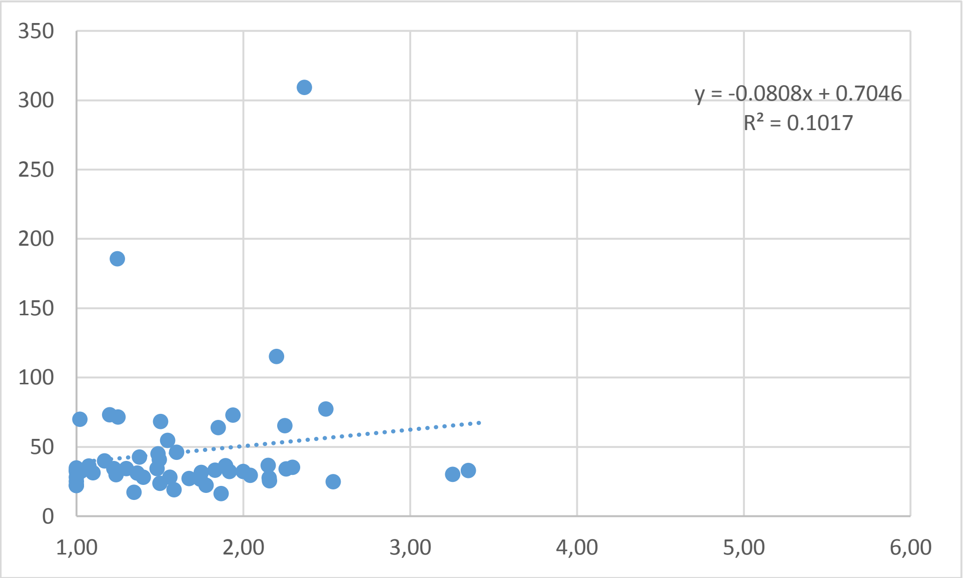

Figure 39: Patients with hypertension who show a normotensive blood pressure (in percentage) and online rating jameda (Indicator 18)

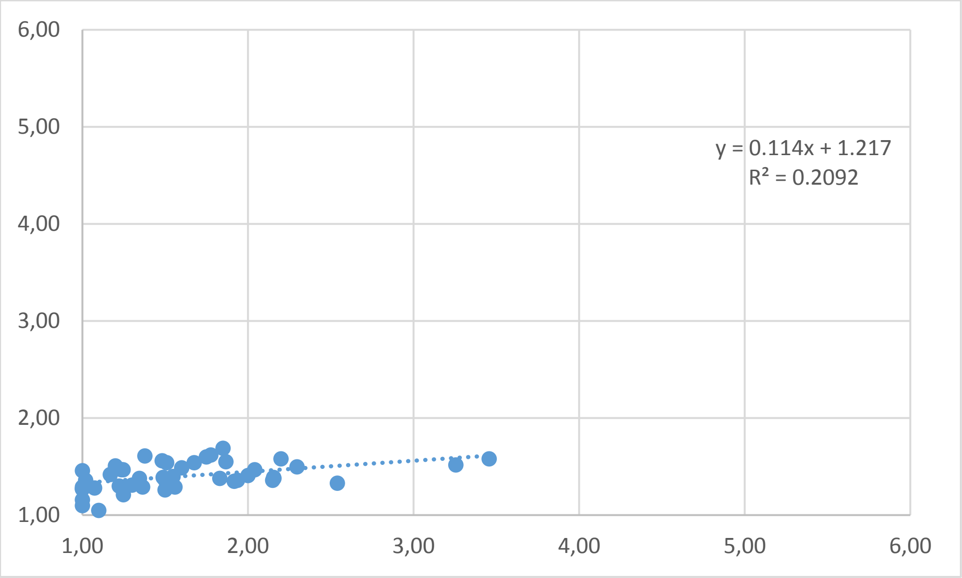

Figure 40: Offline patient survey 2012 (practice-related) and online rating jameda (Indicator 19)

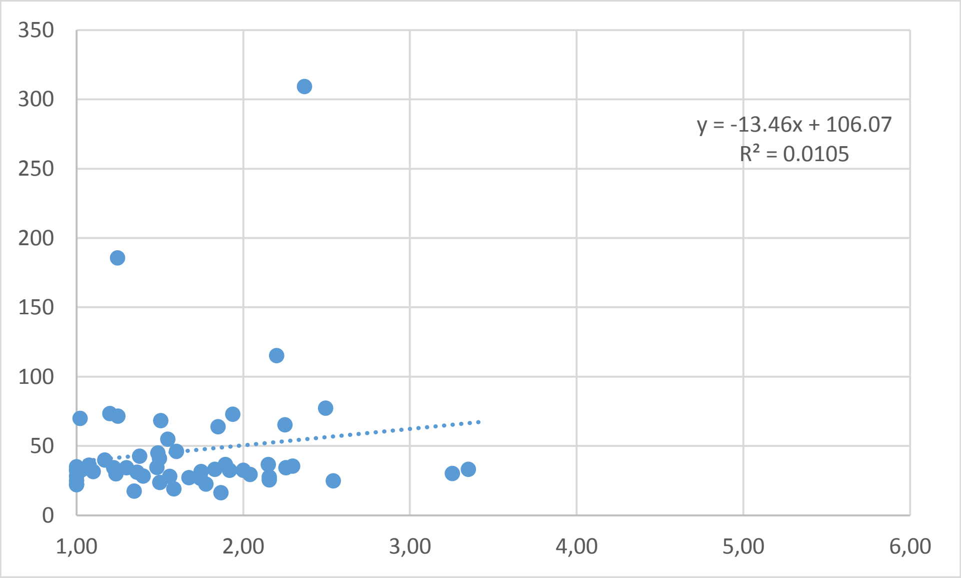

Figure 41: Cost per case (average 2012) and online rating jameda (Indicator 20)

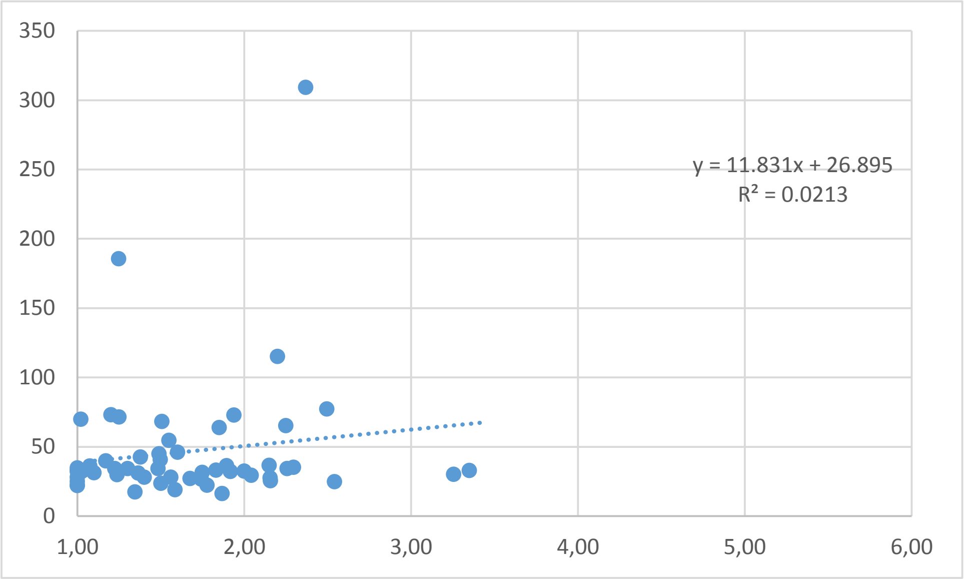

Figure 42: Cost per prescription (average 2012) and online rating jameda (Indicator 21)
